# Correlative montage parallel array cryo-tomography for in situ structural cell biology

**DOI:** 10.1038/s41592-023-01999-5

**Published:** 2023-09-18

**Authors:** Jie E. Yang, Matthew R. Larson, Bryan S. Sibert, Joseph Y. Kim, Daniel Parrell, Juan C. Sanchez, Victoria Pappas, Anil Kumar, Kai Cai, Keith Thompson, Elizabeth R. Wright

**Affiliations:** 1 Department of Biochemistry, University of Wisconsin, Madison, WI USA; 2 Cryo-Electron Microscopy Research Center, Department of Biochemistry, University of Wisconsin, Madison, WI USA; 3 Midwest Center for Cryo-Electron Tomography, Department of Biochemistry, University of Wisconsin, Madison, WI USA; 4 Department of Chemistry, University of Wisconsin, Madison, WI USA; 5 DOE Great Lakes Bioenergy Research Center, University of Wisconsin, Madison, WI USA; 6 Biophysics Graduate Program, University of Wisconsin, Madison, WI USA; 7 Morgridge Institute for Research, Madison, WI USA

**Keywords:** Molecular biophysics, Cryoelectron microscopy

## Abstract

Imaging large fields of view while preserving high-resolution structural information remains a challenge in low-dose cryo-electron tomography. Here we present robust tools for montage parallel array cryo-tomography (MPACT) tailored for vitrified specimens. The combination of correlative cryo-fluorescence microscopy, focused-ion-beam milling, substrate micropatterning, and MPACT supports studies that contextually define the three-dimensional architecture of cells. To further extend the flexibility of MPACT, tilt series may be processed in their entirety or as individual tiles suitable for sub-tomogram averaging, enabling efficient data processing and analysis.

## Abstract (web summary)

Montage parallel array cryo-tomography adopts principles of montage tomography via regular array beam-image-shift montage acquisition and is robust for imaging large fields of view while retaining high-resolution structural information in cryo-electron tomography.

## Main

Cryo-electron microscopy (cryo-EM) of purified proteins (for example, single-particle cryo-EM) has propelled forward the cryo-EM ‘resolution revolution’^1^ resulting in an increasing interest in technologies to enable structure–function studies of macromolecules within the framework of larger biological systems. Cryo-electron tomography (cryo-ET) links three-dimensional (3D) contextual visualization and high-resolution structure determination of cryogenically preserved macromolecules in their native cellular environment^2^. Computationally extracted sub-tomograms can be averaged and classified to reveal sub-nanometer to nanometer resolution (3 Å to 4 nm) structures of in situ complexes^3–5^. Cryo-ET is generally restricted to investigations of small specimen volumes and the thin peripheral areas of cells (<500 nm) that are penetrable by the electron beam. To explore thicker regions of cells, sample thinning technologies have evolved and include cryo-electron microscopy of vitreous sections^6^ and cryo-focused-ion-beam (cryo-FIB) milling^7^, each of which may introduce artifacts to the sample. Cryo-correlative light and electron microscopy (cryo-CLEM)^8^ directly connects temporal and spatial information from fluorescence light microscopy (FLM) with cryo-ET ultrastructural data of a region of interest (ROI). In combination with cryo-FIB milling, it is now possible to pinpoint an area of interest deep in the interior of a specimen with 3D correlative cryo-FLM-FIB-ET^9,10^. However, difficulties remain for bridging the disparity of spatial scales in multimodal microscopy pipelines where the field of view (FOV) in wide-field FLM can be 10^5^ times (~0.1 to 5 mm) that of an EM image (~200 nm to 800 μm)^11,12^.

In cryo-ET, tilt-series acquisition of an ROI involves incrementally tilting the cryo-preserved specimen along one or two axes^13^ while a series of projection images are captured on a detector. To gather both high-resolution structural information^14^ and overall landscape visualization, tilt series could be collected over the same ROI, under both high and low magnifications. However, this approach may be difficult to implement due to the radiation sensitivity of frozen-hydrated biological materials. The collection dose could be lowered, which may result in insufficient acquisition exposure (< 1 e^−^/Å^2^/tilt), reduce signal-to-background noise ratio, and deteriorate data quality. Advances in camera design have supported the use of larger format detectors for certain applications^15^. But the volume of data being digitized scales considerably with the detector size, thus imposing challenges to hardware and software infrastructure^16^. These technical hurdles and others^17^ have constrained the application of high-resolution cryo-ET to small FOVs and fractional volumes of cells. An approach for obtaining 3D tomograms of larger FOVs without sacrificing resolution is to collect montages of tilt series, where the ROI is sampled by overlapping tiled beam exposures that are computationally stitched together during reconstruction. The development of montage cryo-ET has been limited due to low electron dose restrictions and uneven dose accumulation at overlapping regions between adjacent tiles. To our knowledge, only one montage cryo-ET scheme^18^ has been investigated where the electron dose is spread via hexagonal tiling using a restricted beam size. In the end, the user obtains a single, large, stitched montage tomogram. As noted by the authors, major challenges to the reported scheme included seamlessly joining cryo-EM montage tiles with improved automation^18^; processing large volume stitched tomograms^11,19,20^; and performing sub-tomogram averaging (STA)^18^. While the reported hexagon tiling strategy is elegant, this scheme also suffers from low throughput, tile stitching requirements that need substantial user intervention, large volumes of data and a lack of a streamlined workflow adaptable to variety of cellular samples.

**Fig. 1:**
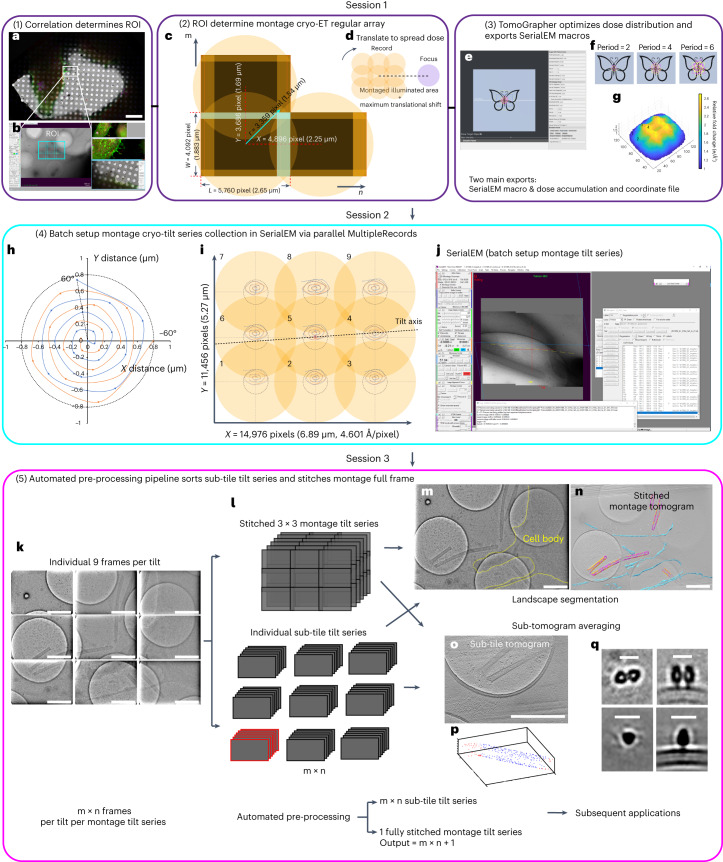
Montage parallel array cryo-tomography methodology. The workflow is broken into three cohesive sessions. Session one (purple boxes, steps 1–3): **a**,**b**, screenshots of CorRelator (**a**) and SerialEM (**b**) to perform accurate on-the-fly FLM to cryo-TEM correlation. **c**, Benchmarked beam tile placement of 15% to 20% in *x* (long axis) and 10% in *y*, optimized on a standard (non-fringe-free) Titan Krios 300 kV microscope with a K3 camera (5,760 × 4,092 pixels) at a pixel size of 4.603 Å. Introduction of translational shifts to the regular array montage pattern (m × n) whose size is rectangular or square, expandable based on the ROI. **d**, Location of focus and tracking area relative to the ‘record’ montaged full illuminated area, for example, 3 × 3 montage collection. **e**–**g**, Application of a simulation from TomoGrapher (**e**) to develop customized montage translational offsets (**f**) in 3D for proper dose distribution (**g**). Session two (cyan box, step 4): **h**, benchmarked spiral translation trajectory applied to distribute the dose. **i**, Cross-correlation alignment of cosine-stretched image shifts at each tilt angle relative to 0° tilt in individual tile tilt series of a regular 3 × 3 montage pattern to ensure ROI retainment, using Tiltxcorr in IMOD. **j**, Screenshot of SerialEM 4.1 during a live montage tilt-series run. Session three (magenta box, step 5): **k**,**l**, individual m × n (3 × 3 = 9) frames per tilt (**k**) were pre-processed via two paths (**l**) to (1) generate a fully stitched FOV and tomogram and (2) individual nine sub-tile tilt series and tomograms, in a total of ten tomograms. **m**–**q**, Subsequent applications for all ten outputs include segmentation (**m**–**o**), image analyses (**o**) and STA (**p**–**q**). Scale bars, 1 μm (**m**–**o**) and 15 nm (**q**).

Here, we developed tools and a workflow to adapt the principles of montage tomography, which is routinely applied to resin-embedded samples^19–21^, to frozen-hydrated specimens via montage parallel array cryo-tomography (MPACT). The MPACT routine and its automated processing schemes (Fig. 1) are robust solutions for the generation of 3D reconstructions of vitrified specimens at molecular-level resolution, with much-increased throughput and adaptability to various sample types. We used a beam-image-shift montage acquisition via parallel illumination strategy to acquire overlapping regular array tile patterns of an ROI. At each tilt, a moderate global translational offset was applied to the pattern to distribute the electron dose. The workflow encompassed three main sessions. First, we used correlative imaging modalities to identify an ROI within a desired larger FOV for montage cryo-ET data collection (Fig. 1a–d). TomoGrapher, an open-source 3D tilt-series simulation tool, was used to optimize tiling strategies and tilt-series collection schemes to spread the electron dose efficiently. Next, the macro exported from TomoGrapher was imported to SerialEM for batch acquisition of montage cryo-tilt series (Fig. 1e–j). Last, tile frames were processed in a streamlined and automated manner, to generate both usable individual tiles and stitched montage tomograms (Fig. 1k,l) for subsequent advanced analyses, such as STA and segmentation (Fig. 1m–q), thereby increasing the throughput and limiting manual intervention. We show the benefits of the MPACT routine for diverse applications, including imaging organellar ultrastructure, imaging budding viral particles, studying cytoskeletal organization in neurites, and STA of filamentous respiratory syncytial virus (RSV) particles.

## Results

### Montage tomography acquisitions and TomoGrapher simulations

**Extended Data Fig. 1:**
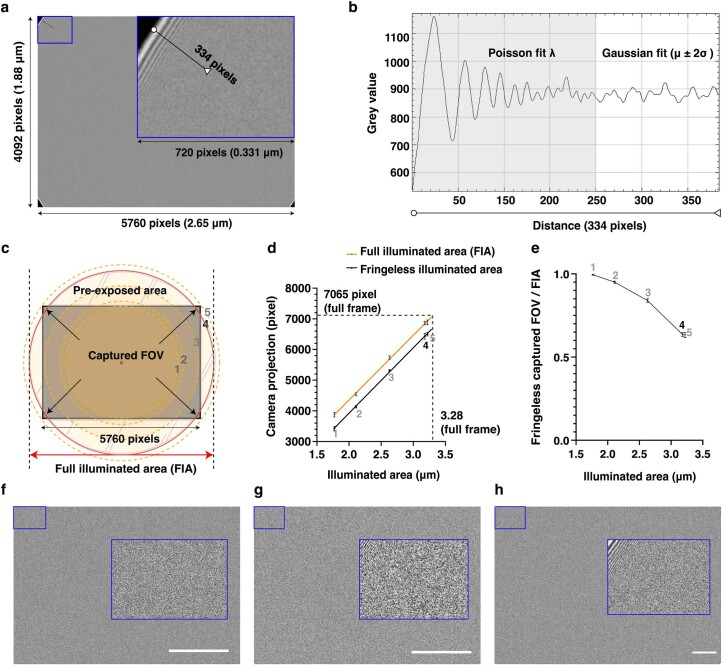
Characterization of beam size and fringe on a triple-condenser lens transmission electron microscope. Benchmarking and optimization of the montage parallel array cryo-tomography (MPACT) workflow was done on a standard Titan Krios 300 kV microscope system with a K3 camera (5760 × 4092 pixels) at pixel size of 4.603 Å, C2 aperture of 100 *μm*, and defocus of −5 μm. **a**. Fresnel fringes extending over the illuminated area (beam size 3.15 *μm*). ***Inset***: Enlargement of the boxed fringe-containing area, low pass filtered to 50 Å. **b**. Radial intensity profile along the black line in the ***Inset*** of (**a**). The region visibly impacted by the Fresnel fringes was determined to extend from the beam edge to ~200 pixels (grey area) towards the center of the image, affecting between 3.5 to 4 % of the outer edge of a tile. Based on the Fresnel fringes’ Poisson behavior and TEM signal’s gaussian distribution, the fringe peak intensities were fit to a Poisson curve (maximum likelihood estimate/peak *λ*), considered as ‘signal’ and the fringeless illuminated area was fit to a gaussian function (*μ* ± 2*σ*) as ‘noise’ in MatLab (*poissfit* and *gaussianFit* Curve Fitting Toolbox). The cut-off from ‘signal’ to ‘noise’ were determined as the possibility of ‘signal’ peaks fading into *μ* ± 2*σ* of ‘noise’ distribution, from multiple measurements (n ≥ 3) along the circular beam edge. **c–e**. Characterization of beam size (1 to 5) in consideration of the full illuminated area (FIA), fringeless illuminated area, pre-exposed area (shaded), and captured field of view (FOV) at the constant benchmark magnification and defocus. Various beam sizes were described by its diameter for simplicity. **c**. The camera FOV (rectangle) and beam size (circles) extending outside the acquired/on-frame area. Beam size decreased from 5 to 1. Beam size 4 of 3.15 *μm* (beam circumference delineated in red) as final optimized beam diameter under the benchmark imaging state, showcases the defined FIA (red horizontal arrow) and captured FOV (four black arrows). See Glossary in the end for reference. **d**. Linear relationships between illuminated areas on a triple-condenser lens Titan Krios system, defined as the electron beam projected onto the sample, and the beam projected size on the camera plane (orange line, orange circles in **c**, *r2* = 0.9989), and the associated fringeless illuminated area (black line, *r2* = 0.9995). **e**. The ratio of fringeless FOV captured on the camera over the full-illuminated area (FIA), following a close linear relationship. **f**. Image of the parallel illumination at the designated ‘fringe-free’ imaging state on a fringe-free-equipped Titan Krios 300 Kv, recorded at the beam diameter of 481 nm that just covers the camera FOV, eucentric focus, 8 e/pixel/s, with a pixel size of 0.6647 Å. No obvious fringes are seen. **g**. Image of the same illumination state as **f** with a defocus of −5 *μm*. Fringes start to show up when the defocus becomes larger. **h**. Image of the parallel illumination at a different magnification (pixel size of 1.689 Å) with a defocus of −5 *μm*, and beam size of 1.19 *μm* that just covers the camera FOV. As the imaging state deviates from the designated fringe-free state, for example, defocus (**g**) and magnification (**h**), the fringes become more obvious on a fringe-free Titan Krios microscope. Scale bars = 100 nm (**f-h**).

**Extended Data Fig. 2:**
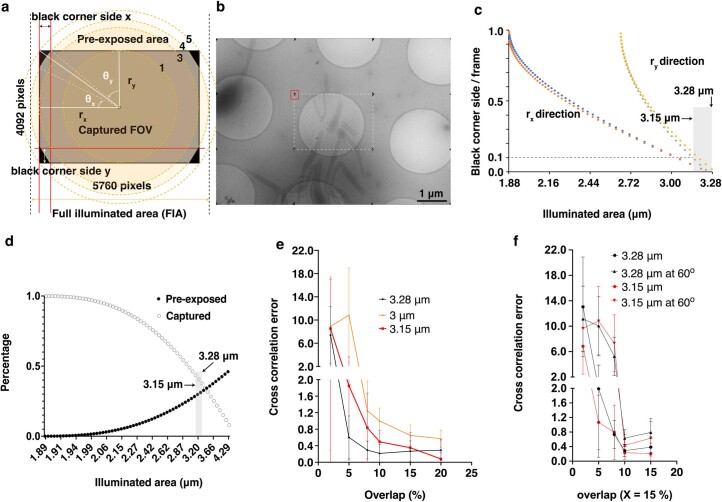
Optimization of beam illuminated area and tiling strategy for montage cryo-ET. **a**. Illustration of change in pre-exposed area, captured field of view (FOV), full illuminated area (FIA), blank/black corners where image information is cut off on both x and y of a frame as the beam size expands and intersects with the full K3 frame’s length (5760, x) and width (4092, y). *θ*_*x*_ and *θ*_*y*_ are the angles between the half frame r_x_ and r_y_ and the beam radius intersecting the frame. The beam size starts from circle 1 where the largest full illuminated area fits into a full camera frame. Both *θ*_*x*_ and *θ*_*y*_ get larger as the beam size expands till the full camera frame into the beam illuminated area, circle 5. **b**. Insufficient overlap between frames and loss of information from four black corners despite the use of a smooth 3 × 3 tile frame stitching. **c**. Ratios of blank/black corner side length in x and y direction against a full K3 camera frame as the beam size expands to include more field of view. The blank/black corner side x (red and blue) starts at the intersecting beam circle 1 (1.88 *μm*) and decreases till the beam circle 5 (3.28 *μm*). Similarly, the intersecting blank/black corner side y (grey/yellow) starts at intersecting beam circle 3 (2.68 *μm*) and decrease till it reaches the circle 5. Red and grey data points represent the blank/black corner side x and y compensated with 4% fringe impact (x*1.04, y*1.04), respectively. **d**. Percentage of pre-exposed and captured FOV against the beam illuminated area. The region between illuminated area 3.15 (circle 4 in a, also benchmark beam size) and 3.28 (circle 5 in a) minimize the blank/black corner in x and y to extend a total montage field of view while preserving a larger captured over pre-exposed FOV to balance undesired dose damage. **e–f**. Cross-correlation error values calculated to stitch a 3 × 3 tile pattern montage of a vitrified sample grid, captured via various beam sizes/illuminated areas, and overlaps in camera full frame length x and y (the same overlap percentage in x and y from 2, 5, 10, 15, 20%) at 0° (**e**, n = 5 independent experiments over 5 RSV virus sites for montage 3 × 3 cryo-ET), and combinations of a fixed overlap in x (15%) with various overlap in y (2, 5, 10, 15, 20%) at 0° and 60° stage tilt (**f**, n = 6 independent experiments over 6 RSV virus sites for montage 3 × 3 cryo-ET). The data were presented as mean ± SD.

We benchmarked the MPACT scheme on a Titan Krios G3i 300 kV FEG-TEM equipped with a rectangular Gatan K3 direct electron detection camera (5,760 × 4,092 pixels), using an imaging state (4.603 Å per pixel in EFTEM mode, defocus range of −3 to −6 μm) typical for cryo-ET and subsequent STA processing. We examined the performance of different beam sizes and overlaps between tiles for dose distribution and automated montage stitching. Specifically, rectangular or square regular array (m × n), such as 3 × 3 or 3 × 4, tile patterns with a moderate circular beam size and optimized tile overlaps were chosen to minimize the impact of Fresnel fringes formed inside the detector FOV from the C2 aperture of the electron microscope^22^. Under the benchmarked imaging state, the beam fringe artifact was determined to affect between 3% and 4% of the FOV extending from the outer edge of the beam size that corresponded to a 3.15-μm (diameter) illuminated area (Extended Data Fig. 1a,b) when the outer edge of the beam intersected the rectangular K3 camera at ~4% of its long axis (*x* axis). Under parallel illumination, the beam size relative to the camera frame determined the full illuminated area, full captured FOV and fringe-unimpacted or ‘fringeless’ area (Extended Data Fig. 1c–h). We examined a series of beam projections on the camera to determine the ratio present between the blank/black edge and preexposed areas (gray region; Extended Data Fig. 2a,d). We then tested the upper and lower limits of this illumination range against a set of tile overlaps to identify a beam size that would have minimal stitching errors at both low and high tilts (beam size 4 of 3.15 μm; Extended Data Fig. 2e,f). This illumination condition supported capturing a larger FOV in one camera frame for downstream analysis of both full montage and individual tile tilt series, thus maximizing data collected from each montage acquisition.

**Extended Data Fig. 3:**
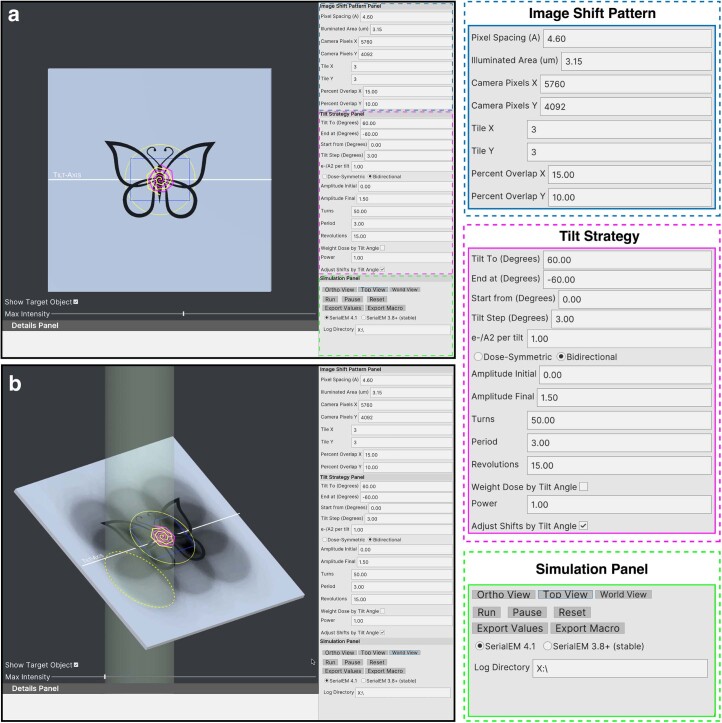
Simulation of tilt series collection in TomoGrapher GUI. *TomoGrapher* is a simulation tool providing a full 3D visualization of **(a)** regular and **(b)** montaged (for example, 3 × 3 tile) tilt series collection. A stage with a target region of interest (ROI, butterfly) shows the beam position relative to the camera frame. The ‘Image Shift Pattern Panel’ (blue box) provides parameters to adjust the pixel size, illuminated area (beam size), camera dimensions, and number of and percent overlap for the montage tiles. The ‘Tilt Strategy Panel’ (magenta box) controls the tilt range, directionality, tilt step increment, dose per tilt, the spiral translational trajectories (yellow, negative tilting angles; pink, positive tilting angles) by which the center of the montage tile pattern follows, during a tilt series. Varying intensity as 1/cosine of tilt angle to a certain power is a selectable option to *Weight Dose by Tilt Angle*. Scaling the y-axis (the axis perpendicular to Tilt axis) shift offset by the cosine of the tilt angle can also be enabled with *Adjust Shifts by Tilt Angle* where the x-axis is aligned as the tilt axis for simplification. The ‘Simulation Panel’ (green box) has buttons to change the viewing angle, start a simulation, export dose accumulation per voxel, and export data including a SerialEM macro to run data acquisition on the microscope.

**Extended Data Fig. 4:**
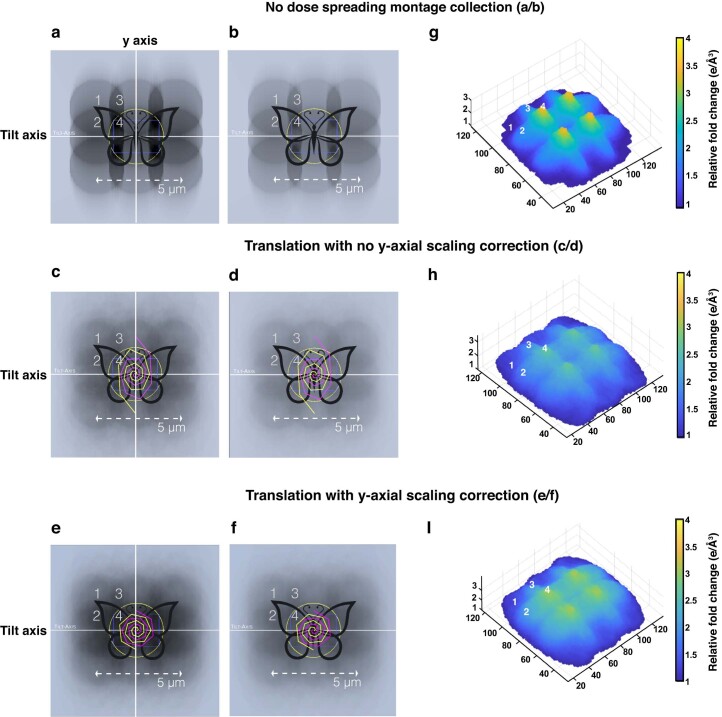
Optimization of montage tiling translational shift for dose distribution in TomoGrapher. Simulated distribution of dose accumulation in six montage tilt series collection schemes (3 × 3 tile pattern, pixel size of 4.603 Å, beam size of 3.15 *μm*, from −60° to 60° tilts with a 3° increment, the butterfly as ROI), with no translational offsets (**a, b, g**), spiral translational offsets (**c, d, h**), and the spiral translational shift with a scaling factor (cosine of tilt angle) to reduce *y*-axis (perpendicular to the tilt axis) stretched shifts at higher tilt angles (**e, f, i**), 1 e/Å^2^ per tile per tilt in (**a, c, e**) and 0.7 e/Å^2^ per tile per tilt in (**b, d, f**). The accumulated dose at areas 1–4 (100 voxels pivoting around the central 1 to 4 points) were compared between the six collection schemes, detailed in Supplementary Table 1. The spiral shift pattern is delineated in pink (0° to 60°) and yellow (−60° to 0°) where the same global translational offset is applied (A_final_ = 2.5, Period = 3, Turns = 50, Revolutions = 15 as parameter inputs controlling the spiral growth). Corresponding visualization of the dose distribution of the ROI butterfly during the tilt series schemes. The maximum (Max) normalized change is ~3.8-fold in (**g**), 2.5-fold in (**h**) and ~2.8-fold in (**i**). Height and color of each voxel correspond to its accumulated dose. The colormap is MATLAB Parula.

**Extended Data Fig. 5:**
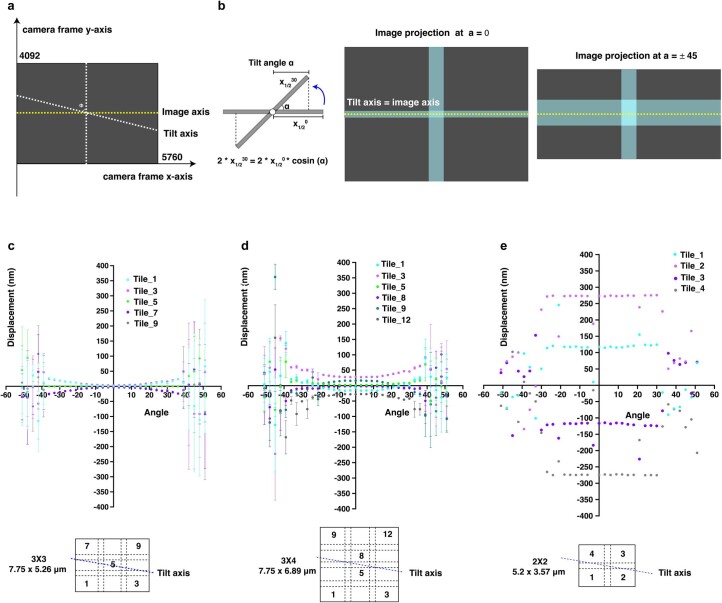
Tiling strategy for montage cryo-ET tilt series collection with global shift offsets to distribute the electron dose. **a**. Schematic of image axis, tilt axis, and tilt axis rotation angles. Image axis x and y generally refer to the cartesian axial system of x and y on the camera frame/image. The tilt axis rotation angle *φ* is labeled when the image axis x/y doesn’t match with the stage tilt angle (tilt axis). **b**. Schematic of changes of projected field of views (xz side view, **b** left; and xy top view, **b** right) and overlap zone (cyan zones in xy top views, **b** right) in the montage cryo-ET tilt series as the stage tilts (tilt angle *α* = 0, *α* = 30°, α = ±45°) during a montage tilt series collection, when the tilt axis matches with the image x axis for simplification (The tilt axis rotation angle *φ* = 0). **c-d**. Calculated initial image shift displacement between successive tilted images of individual tile tilt series at the step of tilt series alignment via *tiltxcorr* (blue dotted line notes the actual tilt axis on the microscope). The montage tilt series (overlap of 15% in X and 10% in Y) were collected using the benchmark imaging condition on a standard Titan Krios 300kV (pixel size of 4.603 Å, C2 aperture of 100 *μ*m, defocus of −5 *μm*, tilt range of −51° to 51°, 3° increment, dose symmetric scheme with group of 3 tilt angles per switch), with an application of the spiral translational shift (3 × 3 in **c** and 3 × 4 in **d**, n = 8 independent experiments over 8 either neurites of primary *Drosophila melanogaster* neurons or RSV virus sites for montage cryo-ET) or the spiral translational plus a 20° per tilt rotational (**e**, total rotation of 35 tilts x 20° = 700°, n = 3 independent experiments over 3 RSV virus sites for montage cryo-ET, mean**)** global offsets for dose distribution. The data were presented as mean ± SD. The montage array pivoted around the center tile by a step of 20° at each tilt angle while shifting along the default Archimedean trajectory (**e**). The addition of rotations to the in-plane image shift caused the displacement and deviation of ROI from both nominal (tilt angle of 0°) and tilt-angle (α) adjusted (y * cos(α)) tiles. This distortion caused automated tile stitching to fail and poor ROI preservation in individual tile tilt series. The benchmark spiral parameters (A_final_ = 1.5, Period = 3, Turns = 50, Revolutions = 15) resulted in ~0.8 *μm* maximum translational offset.

An inherent limitation of montage cryo-ET is the uneven accumulation of dose and possible radiation damage at overlapping regions between adjacent tiles. Therefore, to quantitatively assess cryo-ET data collection and montage tiling strategies, we developed TomoGrapher, a user-friendly simulation tool to visualize tilt-series collection routines and determine global and localized electron dose accumulation per pixel (Extended Data Figs. 3 and 4 and Supplementary Video 1). TomoGrapher used a conventional right-handed coordinate system where the stage canvas is the *xy* plane and the incoming electron beam is directed along the *z* axis (Extended Data Fig. 3a). The ROI (butterfly) in TomoGrapher was tilted around the *x* axis (for example, tilt axis) and identical to an imaging session of the benchmarked Krios TEM. TomoGrapher produced simulations of single or montaged tilt-series acquisitions with and without possible user-defined spiral translation trajectories, with a default setting of an Archimedean spiral included (Extended Data Figs. 3a and 4c–f,h,i and Supplementary Table 1). We tested and adopted a translational shift to the central tile of the montage pattern at each tilt angle (Fig. 1h) to conservatively move the entire montage pattern along a specified trajectory (Fig. 1h,i and Supplementary Video 4). This per-tilt shift distributed excessive dose across the tilt series. During tomography data acquisition, the circular illuminated area and spiral translational trajectory calculated at the 0° tilt angle gradually elongated to ellipses along the *y* axis perpendicular to the tilt axis (*x* axis) as the stage tilts away from 0° (Extended Data Fig. 3b). This behavior resulted in an increase in the total illuminated area and stretching of the spiral paths (Extended Data Fig. 4c,d) when compared to the same exposure dose in an untilted image (Extended Data Fig. 4a,b). Stretching of the spiral paths was corrected by implementing ‘Adjust Shifts by Tilt Angle’ by *y*/*cos α*, where *α* is the tilt angle in the simulation process (Extended Data Figs. 3b and 4e–f). Of note, the geometry of cryo-ET acquisition also caused the ROI located in off-axis areas to move toward the tilt axis *x* (Extended Data Fig. 5a,b) as the stage tilts, following an offset of *y* × *cos α*, where *α* is the tilt angle. Consequently, increased montage overlaps between adjacent tiles were observed in the *y-*axis direction at higher tilts (Extended Data Fig. 5b). To ensure the ROI remained within each tile frame across individual tile tilt series, we used the Multiple Record function in SerialEM^23^ (3.8 and above stable release, and 4.1). This function maintained parallel illumination while adjusting the beam-image shifts per tilt to compensate for off-axis movement. The compensated displacement of the ROI was subsequently examined by measuring the image displacement between successive tilted images within one tile tilt series. The center of an ROI, spiraling along the trajectory in each tile (Fig. 1i), was retained (±200 nm) throughout individual tile/sub-tile tilt series (Extended Data Fig. 5c,d). Accordingly, within the maximum offset distance permitted to retain the FOV in the full tilt range of both tile and stitched montage tilt series (~0.8 μm with the default spiral setting, pixel size of 4.6 Å), simulation results suggested the *y*-axial correction of the spiral pattern introduced more uniformly spread dose (Extended Data Fig. 4i) across each montage tile and tile overlap regions where the accumulated dose would otherwise have been much higher in the absence of any shifts (Extended Data Fig. 4g and Supplementary Table 1). Further comparisons indicated, however, a lower and more restricted accumulated dose in the overlap zone upon the implementation of the stretched ‘unadjusted’ spiral shift (Extended Data Fig. 4h and Supplementary Table 1), likely due to larger translational offsets applied at higher tilts. In practice, we often adopted the *y*-axial tilt angle correction for a more even dose distribution. When the sample is extremely dose sensitive (total dose tolerance lower than 50 e/Å^2^), or area-specific analysis on the tomograms (Extended Data Fig. 10a) is desired, an ‘unadjusted’ translational shift was more beneficial. Nevertheless, the overlap zones always remained in the montage tiling pattern and lowering the per-tile dose was necessary. Empirically, a reduction of 30–40% of the maximum allowable accumulated dose yielded good results.

**Fig. 2:**
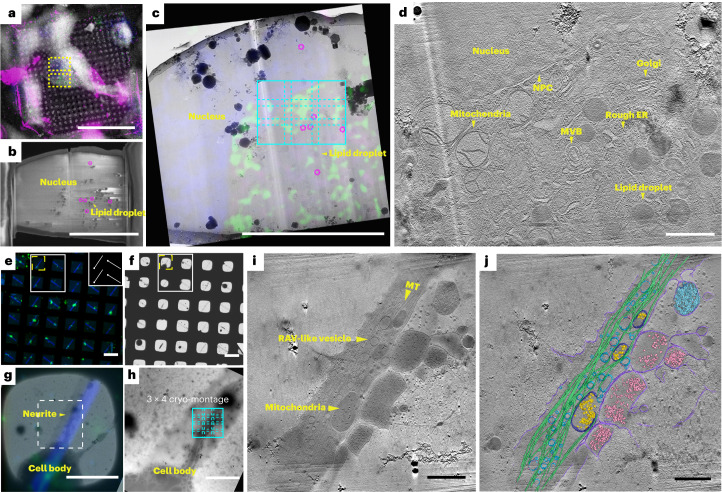
Example applications of correlative cryo-FLM and montage cryo-ET workflows for in situ cryo-lamella of cultured mammalian cells and patterned primary *D. melanogaster* neurons. **a**, Correlative cryo-SEM-FLM to position FIB-milling (yellow boxes) targeting the nucleus (blue) and mitochondria (green) in an A549 cell, with internalized fluorescent beads (40 nm, pink). **b**, Cryo-SEM image of a 200-nm-thick lamella from FIB-milling in **a**. Nucleus, lipid droplets and internalized fluorescent beads (pink circles) are noted. **c**, Correlation of a 2D cryo-EM lamella and the pre-FIB-milled cryo-FLM images to collect a 3 × 3 tile cryo-ET montage (cyan, 6.8 × 5.3 µm at a pixel size of 4.603 Å). **d**, Corresponding tomographic slice, ~45 nm thick (binned 2× tomogram at a pixel size of 9.206 Å) of the cyan ROI in **c**. **e**–**j**, Live-cell FLM (**e**) and cryo-EM (**f**) grid maps of membrane GFP-labeled primary neurons (green) cultured on a straight-line micropatterned grid (inset of **e**) followed by the coating of fluorescent concanavalin A (blue). **g**, Overlay of correlated FLM-cryo-EM image of the square highlighted in yellow in **e** and **f**. **h**, Enlarged cryo-EM image of the dashed white boxed region in **g** where a 3 × 4 tiling for montage cryo-ET (cyan, 6.8 × 6.7 μm at a pixel size of 4.603 Å) collected on the ROI extending from the cell body. **i**,**j**, Tomographic slice of ~45 nm thick (binned 2× tomogram at pixel size of 9.206 Å), reconstructed from the fully stitched montage tilt series at the ROI (**h**) and the corresponding segmentation (**j**). Microtubules (MT, green), surrounding cellular organelles, including mitochondria (dark blue with calcium granules (yellow)), ribosomes (light pink) and ribosome-associated vesicles (RAVs; darker cyan) are noted. Scale bars, 10 μm (**a**–**c**), 1 μm (**d**), 100 μm (**e**,**f**), 50 μm (**g**), 10 μm (**h**) and 1 μm (**i**,**j**).

Together, both simulation results (Extended Data Fig. 4) and experimental data (Fig. 2 and Extended Data Fig. 5c,d) demonstrated that the incorporation of a global translational shift allowed for distribution of the total electron dose across each montage tile and tile overlap regions, consistent with the previous report^18^. Under the benchmarked imaging state, we determined that a 15% overlap in *x* (long axis of a non-square detector) and 10% in *y* (short axis of a non-square detector) of a tile frame was sufficient for automated gap-free tile stitching at each tilt angle without information loss (Extended Data Fig. 2b–f). Occasionally, at higher tilt angles (±48° to 60°), manual adjustment was performed using coordinate-based image cross-correlation in IMOD^24^ when the tile images were of lower contrast with few features or over a grid bar (*n* = 30 of a total of 111, 3 × 3 montage cryo-tilt series).

### Using MPACT with advanced multimodal workflows

**Extended Data Fig. 6:**
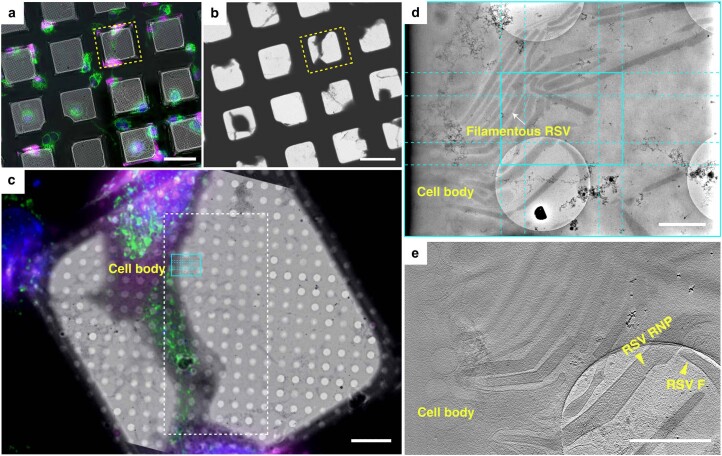
Large field of view of budding RSV virions captured by a correlative cryo-FLM montage cryo-ET workflow. Cryo-FLM (**a**) and cryo-EM (**b**) grid image montages of RSV-infected HeLa cells (magenta) grown on a SiO_2_ filmed R2/2 gold-mesh grid. Mitochondria (green) and nucleus (blue) are labeled. Square of interest highlighted in yellow. **c**. On-the-fly cryo-FLM and cryo-EM 2D correlation in CorRelator (yellow square in **a**, **b**) for the ROI identification where montage tilt series were collected in SerialEM (cyan 3 × 3 tile box). A 3 × 3 tiling for montage cryo-ET was placed at the ROI (cyan tile pattern) where mitochondria cluster and filamentous RSV actively bud from the cell plasma membrane. **d**. Montage cryo-ET tile tilt series at 0° tilt (cyan, 6.8 × 5.3 *μm* at pixel size of 4.603 Å). **e**. Tomographic slice, ~20 nm thick, through the 3 × 3 montaged tomogram (cyan ROI in **c**, **d**). RSV particles with clearly resolved fusion F glycoproteins and ribonucleoprotein (RNP) complexes are present at budding sites along the cell body. Scale bar of 100 *μm* in (**a, b**), 10 *μm* in (**c**), 1 *μm* in (**d, e**).

**Extended Data Fig. 7:**
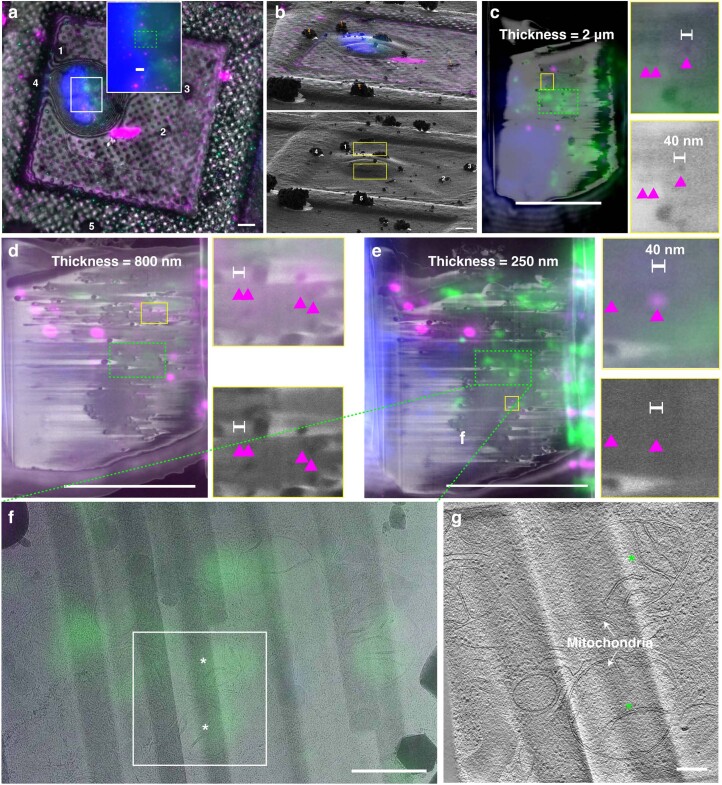
3D correlation using internalized low toxic fluorescent nanoparticles as internal fiducial markers in cryo-FIB-SEM. **a**. Orthogonal merged cryo-FLM (NA = 0.8) projections of labeled mitochondria (green), nucleus (blue), and internalized nanoparticle (pink) within the HeLa cell, acquired via the integrated fluorescence light microscope (iFLM) Aquilos 2 dual-beam system. **b-e**. On-the-fly 3D targeted FIB-milling. The initial x-z correlation of ROI done in 3DCT (registration points 1–4 in **a, b**) and refined x-y plane correlation done in CorRelator (screenshot of correlated FLM-FIB in CorRelator, top, **b**) to guide the yellow milling boxes positioning in z (bottom, **b**). 40 nm nanoparticle intensity visible under cryo-SEM and FLM as the lamella milling progresses from a thickness of 2 *μm* (**c**) to 800 nm (**d**), to 250 nm (**e**), highlighted by pink triangles. **f**. Enlarged overlay of cryo-FLM-TEM of fluorescent signal (green) of mitochondria, corresponding to the green boxed area in (**a-e**). **g**. A central slice of the reconstructed tomogram (thickness of 20 nm, pixel size of 9.454 Å, binned 2, white boxed area in **f**), displaying the targeted mitochondria (asterisks). Scale bars of 10 *μm* in (**a-e**), 1 *μm* in (**f**), 200 nm in (**g**).

**Extended Data Fig. 8:**
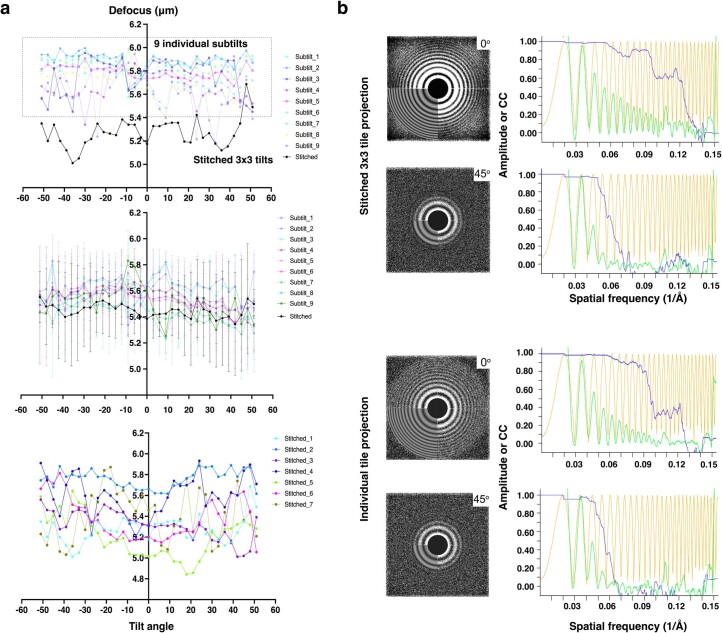
Defocus determination and CTF estimation of full stitched montage and individual tile tilt series. **a**. Defocus values of nine individual tile sub-tilt series and the corresponding stitched 3 × 3 montage tilt series (pixel size of 4.603 Å), were estimated using ctfplotter in IMOD: one exemplary representative 3 × 3 stitched montage and nine individual tile tilt series (top), median variation at each tilt angle over multiple 3 × 3 montages and corresponding tile tilt series (middle, n = 3 independent experiments over 3 montage cryo-ET sites), and stitched montage tilt series (bottom, n = 7 independent experiments over 7 montage cryo-ET sites) are shown. A target defocus of −5 *μm* was applied by performing autofocusing prior to the tile collection at each tilt angle, along the tilt axis, 7.5 *μm* away from the center of the montage tile to account for the size of the montage (6.89 × 5.27 *μm*) and additional translation shift (0.8 *μm*). **b**. CTF estimation in CTFFIND4 by the 2D power spectra (left) from projections at 0°, 45°, and −45° from a representative 3 × 3 stitched montage tilt series and one of the individual tile tilt series (tile position 2 of a regular 3 × 3 tile pattern), and corresponding fitted 1D models (right) showing radially averaged amplitude spectrum (green), CTF fit (orange), and CTF fit score (blue).

We then demonstrated the integration of the MPACT scheme with advanced workflows, such as two-dimensional (2D) and 3D correlative cryo-CLEM and cryo-FIB routines to image along the periphery of HeLa cells (Extended Data Fig. 6) and cryo-FIB-milled lamellae near the nucleus of adenocarcinomatous human alveolar basal epithelial (A549) cells (Fig. 2a–d and Extended Data Fig. 8), respectively. HeLa and A549 cells are commonly used for studies of mitochondria during RSV infection^25,26^. Cryo-CLEM in 2D was applied to identify fields of long, filamentous RSV particles budding from metabolically active RSV-infected HeLa cells^25^ for MPACT data collection (Extended Data Fig. 6a–c). The montage tomogram of RSV particles up to 8 μm in length (Extended Data Fig. 6d) revealed the ultrastructure of intact virions and organization of viral components (Extended Data Fig. 6e). To explore mitochondrial organization closer to the nucleus in naïve or RSV-infected A549 cells, we coupled 3D cryo-FLM-FIB milling (Supplementary Fig. 1 and Extended Data Fig. 8) and targeted fluorescently-labeled mitochondria-rich areas near the nucleus where multiple 3 × 3 montage tilt series were collected via MPACT (Fig. 2a–d), each montage covering an area of ~7 × 5.5 μm^2^ (representative 3 × 3 montage tile in cyan box; Fig. 2c) in contrast to a single frame of ~2.6 × 1.8 μm^2^. Low-toxicity fluorescent nanoparticles (40 nm in diameter)^27^ were internalized by the cells and used as FIB-milling ‘fiducials’ to position and adjust milling boxes on the fly in the *z* and *xy* planes based on the position of nanoparticles relative to the ROI in the 3D cryo-FLM *z*-stack (Supplementary Fig. 1). A square of interest was identified (Fig. 2a) and milling boxes were initially positioned via external markers using 3DCT^9^ and CorRelator^28^ (Fig. 2b, Supplementary Fig. 1 and Extended Data Fig. 7a,b), then further adjusted based on the FIB-milling ‘fiducials’ (Extended Data Fig. 7c–e) during the thinning process. The nanoparticles, uniform in size and density, were readily differentiated from electron-dense lipid droplets and ice crystals under cryo-scanning electron microscopy (cryo-SEM; Fig. 2b and Extended Data Fig. 7c–e) and cryo-ET. The post-correlation on-lamella cryo-EM or cryo-SEM workflow with corresponding cryo-FLM sections confirmed the presence of nanoparticles and success in relocating the ROI (Fig. 2b,c and Extended Data Fig. 7f). Overall, improved 3D correlative targeting in combination with MPACT supported the precise acquisition of large FOVs of an FIB-thinned cellular lamella. Within the 3D volume, the ultrastructure and arrangement of mitochondria, Golgi, rough endoplasmic reticulum and nuclear pore complexes along the nuclear envelope were observed (Fig. 2d and Supplementary Video 2).

Next, we applied the MPACT scheme to primary neurons grown on micropatterned cryogenic transmission electron microscopy (cryo-TEM) grids^29,30^. Coupling micropatterning with cryo-ET has proven valuable for directing cytoskeleton organization^30^ and understanding neural outgrowth^29^. Straight-line patterns were used to control neurite growth of primary *Drosophila melanogaster* neurons from the brains of third-instar *D. melanogaster* larvae (Fig. 2e–h). Multiple montage tilt series were collected along neurites protruding from peripheral areas of the cell body (representative 3 × 4 montage site (~8 × 7 μm^2^) delineated in Fig. 2h). The montage cryo-tomogram revealed the architecture of the cytoskeleton (Fig. 2i,j), including continuous microtubules (~9 μm) stretching along the pattern and the presence of actin filaments extending from the neurite (Supplementary Video 3). The MPACT strategy is important for designing flexible acquisition strategies to capture large-scale 3D molecular vistas of neurons and other cells that are responding to external physical cues such as those imposed by micropatterning.

### Sub-tomogram averaging combined with MPACT

A fully reconstructed unbinned stitched 3 × 4 montage tomogram could be ~700 GB or more, particularly when the sampling pixel size decreases and tile pattern size expands. To maximize output and develop efficient STA processing schemes, we sorted individual tile tilt series and reconstructed them into tile tomograms. Consistent with simulation results (Extended Data Figs. 3 and 4), each tile tilt series exhibited the same spiral image-shift trajectory as the fully stitched montage tilt series (Fig. 1i and Supplementary Video 4). Restricting the largest image translation offset to 30% or less of the FOV per image (Fig. 1h) maintained the ROI within individual tile tilt series during tilting. After determining the eucentric plane of the montage field, autofocus and drift correction were performed at each tilt angle using an area away from the center tile to protect the ROI along the tilt axis (Fig. 1c,d) during dose-symmetric acquisition^31^. This same area was used for tracking the ROI in both tilt directions. While per-tile defocus adjustment was not applied, contrast transfer function (CTF) determination using strip-based CTFPLOTTER^32^ indicated that the MPACT scheme provided a relatively stable defocus (±1 μm) over tilt series acquired from a wide range of samples (Extended Data Fig. 8a). We used whole-image-based CTFFIND4 for tilted defocus determination^33^. CTFFIND4 reported the high-resolution limit of detected Thon rings for individual tiles and stitched montages at 0° tilt (8 Å) and high tilts (20 Å; Extended Data Fig. 8b) at the pixel size of 4.6 Å. In future developments, per-tile defocus adjustment could be used for larger tile arrays and when FIB-milled lamella are deformed^34^.

**Extended Data Fig. 9:**
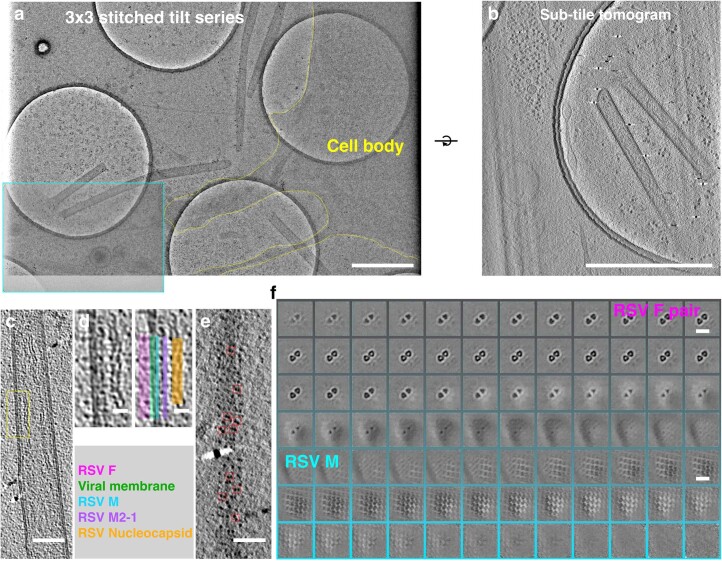
Sub-tomogram averaging of RSV F pair using individual sub-tile tomograms from 3 × 3 montage cryo-ET collection. **a**. A stitched 3 × 3 MPACT tilt series image of RSV-infected BEAS-2B cells at 0°. The cell boundary is delineated in yellow, and one sub-tile tilt series is highlighted in cyan. **b**. Central ~18 nm slice of the tomogram rotated 90° around x-axis to preserve the handiness of the sub-tile tomogram in (**a**, cyan box, pixel size of 9.206 Å) where filamentous RSV particles were preserved. **c–e**. Reoriented Z-projection (9 nm thick) of the virion from the sub-tile tomogram in (**b**) where enlarged views of the virus (**d**) display clearly various virion components noted in different colors, and € the RSV glycoprotein F protein pairs on the surface, viewed looking towards the membrane from the outside, picked for sub-tomogram averaging. **f**. Volume slices (128 × 128 × 128 pixel^3^) through the F-pair sub-tomogram average filtered to 22 Å from individual tile tomograms (pixel size of 4.603 Å, n = 13021), showing the top F-pair (grey boxed) and bottom M matrix layer (cyan boxed). Scale bars of 1 *μm* in (**a**, **b**), 100 nm in (**c**) and (**e**), 20 nm in (**d**), 15 nm in (**f**).

**Extended Data Fig. 10:**
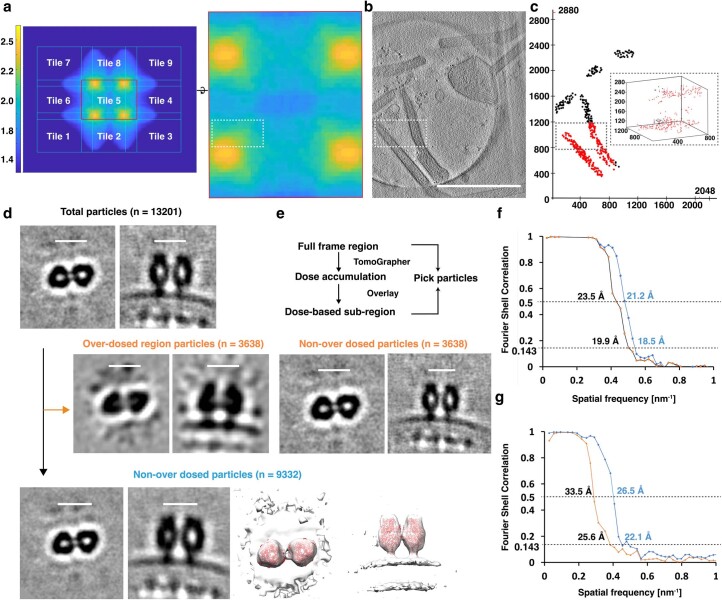
Impact of over-dose region particles on sub-tomogram averaging using individual sub-tile tomograms. **a**. 3D plot (view in from the top, xy plane) of the dose accumulation and distribution simulated profile of a benchmarked 3 × 3 MPACT tilt series (±60°, increment of 3°), exported from the *TomoGrapher* simulation. Each of the sub-tiles (Tile 1–9) were sorted and reconstructed independently for subsequent RSV F-pair particle picking and sub-tomogram averaging. Using the central sub-tile (Tile 5) as an exemplary representative here, particles within two-fold and above dose-accumulated regions of individual sub-tile tomograms were excluded. **b-c**. Visualization of the coordinates of cropped RSV F-pair sub-tomograms on one exemplary representative central Tile 5 tomogram. Particle coordinates within the over-dosed regions delineated in red, and outside the over-dosed regions in black. The magnified 3D view of over-dosed (red) and non-over-dosed (black) RSV F-pair within the dashed boxed region in the coordinate graph (**c**), tomographic slice (white box, **b**) and simulation map (white box on the central tile 5, **a**, right). The same region-based particle sorting was applied to all individual sub-tile tomograms (n = 20) collected via 3 × 3 MPACT. **d**. Sub-tomogram averages of RSV F-pairs viewed from the top (left) and rotated 90° to the side (right), from all regions within the twenty sub-tile tomograms (top panel, RSV F-pair particle number n = 13021, filtered to 24 Å), and the two-fold and above over-dosed regions (middle panel, left: n = 3689, filtered to 34 Å), the region outside of the over-dosed areas with the same number of particles (middle panel, right: n = 3689, filtered to 27 Å), and total particles from the regions outside of the over-dosed areas (bottom panel, left: over-dosed particles excluded, n = 9332, filtered to 22 Å). Isosurface rendering of the sub-tomogram average in (**d**. bottom panel, left) with models of RSV F (PDB: 4JHW, red) fit into the density. **e**. Workflow diagram of picked particle sorting based on region and associated dose accumulation per voxel from *TomoGrapher*, to determine over-dosed particle coordinates. **f**. Un-masked correlated FSC curve showing a global resolution of 21.2 Å and 18.5 Å for the over-dosed removed RSV F-pair sub-tomogram average (blue line, **d**, bottom panel), and 23.5 Å and 19.9 Å for all picked RSV F-pair sub-tomogram average (black line, **d**, top panel), at the FSC cutoff of 0.5 and 0.143, respectively. **g**. Un-masked correlated FSC curve showing a global resolution of 33.5 Å and 25.6 Å for the over-dosed RSV F-pair sub-tomogram average (orange line, d, middle panel left**)**, and 26.5 Å and 22.1 Å for over-dosed removed RSV F-pair sub-tomogram average (blue line, **d**, middle panel right) with the same number of randomly picked particles (n = 3638), at the FSC cutoff of 0.5 and 0.143, respectively. All RSV F-pair sub-tomogram averages at the unbinned pixel size of 4.603 Å, calculated via Gold Standard *SimpleFSC* in PEET. Scale bars, 1 *μm* in (**b**), 10 nm in (**d**).

RSV fusion (F) glycoproteins are arrayed in pairs^35,36^ on the surface of budding or released filamentous RSV particles (Extended Data Fig. 9a–e). We performed STA on RSV fusion (F) glycoprotein pairs to compare with reported observations^36,37^. Volumes containing both F-pairs (top) and matrix (M) protein layer (bottom) were extracted from the individual tile/sub-tile tomograms and averaged to reveal 3D structural features (Extended Data Fig. 9f; *n* = 13,021, 19.9 Å at the Fourier shell correlation (FSC) cutoff of 0.143, no symmetry imposed). To determine if improved averages would result from when particles located in overdosed zones (~twofold above the regular dose tolerance) were removed from the average (Extended Data Fig. 10a), 3,689 F-pairs from those regions (overdosed particles in red from one exemplary tile tomogram; Extended Data Fig. 10b–c (red coordinates); resolution of 25.6 Å at FSC cutoff of 0.143) of the 13,021 total unique particles (Extended Data Fig. 10d; resolution of 19.9 Å at FSC cutoff of 0.143) were removed to yield a refined F-pair average at ~18.5 Å resolution (at FSC cutoff of 0.143; Extended Data Fig. 10d; *n* = 9,332). Overdosed F-pairs showed obviously deteriorated structural features (Extended Data Fig. 10d), when compared to the non-overdosed average (Extended Data Fig. 10d). The averaged F-pair structures were on par or better than what has been reported^36,37^ using a moderate pixel size of ~4.6 Å. We anticipate further improvements to an average could be gained by using a smaller pixel size, the incorporation of a larger number of particles and further refinement of per-particle CTF correction^38^.

## Discussion

In conclusion, the MPACT scheme presented in this study is suitable for capturing comprehensive fields of view of targeted regions of interest in complex biological environments at molecular-level resolution. To meet the increasing demand for high throughput cryo-ET, we demonstrated that this montage cryo-ET approach yields both fully stitched montage reconstructions for more comprehensive information, and m × n usable individual tile reconstructions for STA. It is noteworthy that MPACT is applicable for TEMs with and without fringe-free optical states, and is adaptable to existing imaging routines, thus supporting flexible tile patterns, streamlined batch tilt-series acquisition, and automated stitching and processing. There have been challenges associated with implementing montage cryo-tomography for thicker samples or those with a poor signal-to-background noise ratio^18^. These were partially due to restricted beam sizes, loss of captured FOVs at each camera frame and requirements for manual tile stitching. In addition, all montage cryo-ET schemes, even with the application of dose spreading routines, still introduce uneven and additional exposures across overlap areas. This necessitates a reduction of data collection dose by 30–50% and the proper application of dose weighting^39^. It is possible that the inclusion of apertures of different geometries^40^ could offer some benefits by extending the FOV within a frame or partially reducing overlaps. However, to seamlessly fully stitch tomography montages over standard tilt ranges, sufficient and generally >10% overlaps for cryo-ET are required. Notably, the exposures along the direction perpendicular to the tilt axis increasingly overlap with either square or regular round beams. Thus, proper dose distribution strategies must be considered to minimize nonuniform exposures in the overlapped zone (Extended Data Fig. 4). As pointed out^40^, a new data acquisition scheme will be necessary to take advantage of the ‘square beam’ for large FOV cryo-ET applications. Another advantage of MPACT is the retention of a stable defocus value across all tiles, such that precise defocus determination can be estimated. Further refinements may be applied to each tile of moderate or large tiling pattern dimensions by using a geometric relationship^34^ for per-tile prediction-based defocus adjustments.

Individual tile tilt series may be extracted from the full montage, processed and used for STA workflows. This modified pipeline reduces computational overhead, enables dose-weighted particle sorting based on coordinate-based location and supports reliable CTF estimations per tile tilt series in sub-tomogram averages. In real time, the data collection and processing routines can be tested and improved by using the simulation tools in TomoGrapher where the coordinate location and dose accumulation profile is generated. As a result, careful tilt series processing and particle selection allow for the routine generation of nanometer to sub-nanometer native structures by STA. This offers a streamlined alternative for a recently published hexagonal-packing montage cryo-ET scheme where the storage of large datasets and cross-platform framework processing pipelines^41^ are required for high-resolution STA. Furthermore, each macromolecular structure from a sub-tomogram average or contained within an individual tile tomogram may be ‘mapped back’ to its coordinate position in the full montage tomogram thereby supporting a more comprehensive and contextual understanding of macromolecules within the cellular confines.

MPACT could also lay the foundation for future developments with super-montage tomography that incorporate both image-shift and stage-shift collection strategies^21^. Ultimately, montage tomography solutions will help bridge the resolution gap and FOV losses present between multimodal microscopy imaging pipelines.

## Methods

### Cell lines and cell culture

HeLa cells (American Type Culture Collection (ATCC) CCL-2) and A549 cells (ATCC, CCL-185) were cultured and maintained in supplemented DMEM complete medium and BEAS-2B cells (ATCC, CRL-9609) were cultured in supplemented RPMI-1640 complete medium as reported previously^25^. Primary *D. melanogaster* third-instar larval neurons (the strain elaV-Gal4, UAS-CD8::GFP maintained and kindly provided by the Wildonger laboratory, University of California, San Diego) were extracted, cultured in supplemented Schneider’s medium and maintained on micropatterned grids as previously described^42^.

### Cell seeding, infection and in situ labeling on TEM grids

Cell seeding on the TEM grids was performed following previous reports^25^. Briefly, Quantifoil grids (200 mesh gold R2/2 carbon or silicon dioxide film; Quantifoil Micro Tools) were coated with extra carbon (5 to 8 nm) and glow discharged (10 mA, 60 s). HeLa and BEAS-2B cells were trypsinized and seeded at a density of 0.5–0.7 × 10^5^ cells per milliliter, followed by an overnight incubation before subsequent applications. For montage cryo-ET and correlative cryo-FLM-montage-ET, HeLa and BEAS-2B cells on the grids were infected with the recombinant virus strain RSV rA2-mK^+^ at an optimized multiplicity of infection of 10 for 24 h at 37 °C and 5% CO_2_, as determined previously^25^. For cryo-focused-ion-beam milling (FIB-milling), A549 cells were digested and seeded at a density of 0.3 × 10^5^ cells per ml on Quantifoil grids (silicon dioxide, gold) for 16 to 24 h.

### Micropatterning and neuron cell culture on TEM grids

Micropatterning and culturing of primary *Drosophila* larval neurons were performed as described^29^. Briefly, the extra carbon-coated gold Quantifoil grids (200 mesh, R 1.2/20, holey carbon film; Quantifoil Micro Tools) were glow discharged and coated with 0.05% poly-l-lysine. The grids were then functionalized by applying a layer of anti-fouling polyethylene glycol-succinimidyl valerate, followed by application of a photocatalyst reagent, 4-benzoylbenzyl-trimethylammonium chloride gel. Maskless photopatterning was performed to ablate the anti-fouling layer in defined patterns with an ultraviolet laser (*λ* = 375 nm, at a dose of 30 mJ/mm^2^) using an Alvéole PRIMO micropatterning system. Adherent extracellular matrix protein, fluorescently-labeled concanavalin A, Alexa Fluor 350 conjugate (emission, *λ* = 457 nm, 0.5 mg ml^−1^ in water or PBS, Thermo Fisher Scientific) was then added to promote the cellular adhesion and growth of primary *Drosophila* larval neurons isolated and cultured according to established protocols^42,43^. These neurons had pan-neuronal GFP expression (emission, *λ* = 525 nm) on the membrane (CD8-GFP) to allow for tracking using live-cell wide-field fluorescence microscopy imaging. Neurons on patterned grids grew for a minimum of 48–72 h for neurite growth before plunge freezing.

### Vitrification

For EM grids prepared for non-FIB cryo-ET applications, 4 μl of 10 nm BSA-treated gold fiducial beads (Aurion Gold Nanoparticles, Electron Microscopy Sciences) were applied before vitrification. The grids were plunge frozen using either a Gatan CryoPlunge3 system (CP3) with GentleBlot blotters (Gatan) or a Leica EM GP (Leica Microsystems). The Gatan CP3 system was operated at 75–80% humidity and a blot time of 4.5 to 5.5 s for double-sided blotting and plunge freezing. The Leica EM GP plunger was set to 25 °C to 30 °C, 99% humidity and blot times of 6 s for R 1.2/20 micropatterned carbon-foil grids, and 15 s for R2/2 silicon dioxide foil grids for single-sided back blotting and plunge freezing. Plunge-frozen grids were then clipped and stored in cryo-grid boxes under liquid nitrogen.

### Correlative live-cell and cryogenic fluorescence microscopy

Uninfected A549 or RSV-infected HeLa cells were stained with MitoTrackerGreen FM (M7514, Thermo Fisher Scientific, 100 nM, 30 min at 37 °C and 5% CO_2_), washed and stained with Hoechst 33342 (H3570, Thermo Fisher Scientific, 1:1,000 dilution, 20 min at 37 °C and 5% CO_2_) to visualize mitochondria and the nucleus. As reported previously^8^, live-cell wide-field imaging (×20, 0.4-NA lens, dry) and cryo-FLM (×50, ceramic-tipped, 0.9-NA lens) on vitrified samples were performed on a Leica DMi8 and Leica EM Cryo-CLEM THUNDER system, respectively. On the DMi8, the bright-field and bandpass filter cubes of FITC (emission, *λ* = 527/30 nm), DAPI (emission, *λ* = 460/50 nm), TXR (emission, *λ* = 630/75 nm) and Y5 (emission, *λ* = 700/75 nm) were used. On the Cryo-CLEM THUNDER system, the bandpass filter cubes used included GFP (emission, *λ* = 525/50 nm), DAPI (emission, *λ* = 460/50 nm), TXR (emission, *λ* = 630/75 nm) and YFP (emission, *λ* = 630/75 nm). Live-cell wide-field images were collected as a grid montage at ×20. For cryo-FLM, *z*-stack projections of 12 to 15 μm for each channel were collected on the vitrified sample at a Nyquist sampling step of 350 nm using the Leica LAS X software. Small Volume Computational Clearance from the Leica LAS X THUNDER package was applied for fluorescence image deconvolution and blurring reduction on the cryo-FLM image *z*-stacks. All images and mosaics were exported and used as LZW compressed lossless 16-bit TIFF format. On-the-fly cryo-FLM to cryo-ET correlation and data collection were performed using CorRelator^28^. Grids that were imaged under cryogenic conditions were saved and stored in cryo-grid boxes under liquid nitrogen.

### Three-dimensional targeted Cryo-FIB-SEM

Low-toxicity nanoparticles of 40 nm (FluoSpheres, carboxylate modified microsphere, dark red fluorescent (660/680 nm), Thermo Fisher Scientific, F8789) were introduced to cells seeded on the grid for an incubation of 2 h at 2 mg ml^−1^, followed by washing with 1× PBS and 5 min incubation of 5–10% glycerol as a cryoprotectant to properly vitrify cells. Afterwards, 4 μl of diluted 200 nm (1:200 dilution, dark red fluorescent (660/670 nm), Thermo Fisher Scientific, F8807) or 1-μm FluoSpheres (1:500 dilution, red fluorescent (585/608 nm), Thermo Fisher Scientific, F13083) were applied to the grid in the humidity chamber of the Leica EM GP plunger before the vitrification step. Following imaging using a Leica EM Cryo-CLEM THUNDER microscope, clipped grids were transferred into a dual-beam (SEM/FIB) Aquilos 2 cryo-FIB microscope (Thermo Fisher Scientific) operating under cryogenic conditions. To improve the sample conductivity and reduce the curtaining artifacts during FIB-milling, the grid was first sputter coated with platinum (10 mA, 15 to 30 s), and then coated with organometallic platinum using the in-chamber gas injection system (GIS, 75 s with a measured deposition rate of 60 nm s^−1^). A 2D affine transformation on the *xy* plane was performed to align cryo-FLM and cryo-SEM grid mosaics on a rough micron scale and to further correlate square images from two modalities on a fine nanometer scale precision using hole centroids or 1-μm FluoSpheres in CorRelator. The eucentric height of the ROI on the cryo-shuttle inside the dual-beam microscope and a shallow FIB-milling angle of 8° to 12° were determined. SEM and FIB 2D views of the squares (with the FOV large enough to include sufficient external microspheres and features as registration points) that contained the ROI were collected at the eucentric height and milling angle. A 3D coordinate transform between the 3D *z*-stack of the Y5 channel (nanoparticles, emission *λ* = 680 nm) and the 2D FIB view was conducted through the optimized rigid body 3D transformation algorithm available in the 3DCT package using external FluoSpheres (*n* = 4 to 10) as registration points^9^. The transformed coordinates (*x*, *y*, *z*) were then imported into CorRelator^28^ to fine tune the deviations in *X* and *Y* coordinates introduced by the *Z* transformation in 3DCT, using the closed-form best-fitting least-square solution. The FIB-milling boxes were positioned based on the prediction in the 2D FIB view in CorRelator. The ion-beam milling process was performed using 0.3 nA for rough milling and gradually decreased currents of 0.1 nA, 50 pA, 30 pA and 10 pA, following previously established protocols^44^. Without changing the sample/shuttle position during the milling, a series of cryo-SEM images (electron beam set at 2 kV, 25 pA, dwell time of 1 μs) were collected as the lamella was thinned from an initial thickness of 5 μm, 3 μm and 1 μm, to 800 nm, 500 nm and to the final 200 nm. The SEM images were used to: (1) check the milling process related to stage drift, lamella bending and so on; (2) adjust the milling positions by visualizing the density of internalized 40-nm nanoparticles on the lamella and comparing their positions (*x*, *y* in ~100 to 200 nm deviation error, and 500 nm in *z* relative to the ROI) in the correlated FLM *z*-stack; and (3) confirm the successfully milled isolated region houses the ROI. On-the-fly monitoring of nanoparticle presence provided quick and movement-free feedback on 3D targeted milling when an integrated FLM system is not available. It could also help eliminate excessive alignment steps introduced by the shuttle moving in an integrated FLM and FIB-SEM system when the sample is moved back and forth between the FLM imaging and FIB-SEM positions in most of the current integrated FLM-FIB/SEM systems^45–47^.

To further confirm the preservation of an ROI in the lamella, lamellae were transferred to the Leica EM Cryo-CLEM THUNDER^28^ or Leica Stellaris cryo-confocal system and a *z*-stack of the same channels was collected. With the post-correlation on-lamella cryo-FLM workflow, cryo-SEM and cryo-EM were performed using angle-corrected neighboring fluorescence signals around the lamella to transform cryo-FLM signals to corresponding features on the lamella as described previously^48^. If the lamella was made in an integrated FLM-cryo-FIB Aquilos 2 microscope (Thermo Fisher Scientific), the corresponding *z*-stacks were directly collected via the integrated FLM objective (×20, 0.85 NA) inside the FIB/SEM chamber to validate the presence of ROI and nanoparticles throughout the milling process.

### Cryo-electron tomography and reconstructions

After live-cell FLM, cryo-FLM imaging and/or cryo-FIB milling, the same clipped frozen grids were imaged using a Titan Krios G3i (Thermo Fisher Scientific) at 300 kV without the fringe-free optical state. Images were acquired on a Gatan Bioquantum GIF-K3 camera (Gatan) in EFTEM mode using a 20-eV slit. Images were captured at various magnifications of ×81 (4,485 Å/pixel) for whole-grid mosaic collection, ×470 (399 Å per pixel) and ×1,950 (177.6 Å per pixel) for square or whole lamella overview, ×6,500 (27.4 Å per pixel) for intermediate magnification imaging where the FOV is suitable for reliable tracking and 40-nm nanoparticles are visibly distinguished, and ×19,500 (4.603 Å per pixel) for data acquisition using the SerialEM software (v.3.8.7) package^23^. Fresh correlated-double sampling mode (CDS) counting gains with the designated beam size (for example, 3.15 μm) were prepared in the Gatan Microscopy Suite Software. The full frame size of 5,760 × 4,092 pixels (counting, CDS mode at the dose rate of 10 e^−^pixel^−1^s^−1^, bin2 at hardware in SerialEM) was collected.

#### Montage cryo-ET setup in SerialEM

Regular image-shift montage acquisition and the multiple record function in SerialEM^23^ (v.3.8.7 64-bit package or v.4.1 beta, for example, 4.1.0 beta11 64-bit package where the Multiple Record Montage is included) were adapted for implementation of overlapped beam-image-shift tiling. Benchmarks were done at a data acquisition magnification (pixel size of 4.603 Å per pixel) typical for cryo-ET. The illuminated area of 3.15 μm in diameter on the sample was determined by the beam size on the camera and the lens magnification. Fresh gains were collected with this beam size (3.15 μm) and the gain normalized image over vacuum was low-pass filtered to 50 Å to enhance the signal of Fresnel fringe peaks. Based on the behaviors of Fresnel fringes^49^ and EM Gaussian signal distribution, the intensity value of the image over pixel was fitted into two distribution curves, a Poisson curve (maximum likelihood estimate/peak *λ*) to fit the edge areas considered as ‘signal peaks’ using 20% of *x* and *y* dimensions extending from the edge toward the center and a Gaussian curve (*μ* ± 2*σ*) to fit the center area considered as noise/background using 90% of whole *x* and *y* dimensions from the center toward the edge with a 10% overlap with the ‘edge’ area in MATLAB (poissfit and gaussianFit Curve Fitting Toolbox, MathWorks). The cutoff from ‘signal’ to ‘noise’ was determined as the possibility of ‘signal’ peaks fading into *μ* ± 2σ of ‘noise’ distribution. From multiple measurements (*n* ≥ 3) along the circular beam edge, the cutoff was 3.5–4% of *x* extending from the edge and an unmeasurable amount in the *y* direction. Thus, the rest of the image was considered as a fringeless FOV. The FOV affected by Fresnel fringes becomes more evident with a linear decrease in beam size. Over a wide range of samples, we selected the pixel overlaps of 15% to 20% in the long-axis *x* direction and 10% in the short-axis *y* direction as the optimal tiling strategy, such that the usable FOV for each camera frame was maximized after correction of Fresnel fringes and optimization of the preexposed area outside the camera frame FOV. Additionally, a Titan Krios G3i TEM (Thermo Fisher Scientific) operated at 300 kV under ‘fringe-free’ conditions was used to define the C2 aperture-derived residual fringes as the beam size, magnification and defocus deviated from the specific ‘fringe-free’ illumination condition in EFTEM mode (beam size of 481 nm, magnification of ×165,000 with a pixel size of 0.6647 Å, eucentric/standard focus determined by the manufacturer) on a Gatan Bioquantum GIF-K3 camera (Extended Data Fig. 1f–h).

A rigorous and reliable image-shift calibration in SerialEM at the data acquisition magnification was performed beforehand and repeated to ensure a more accurate shift. In SerialEM (stable release, v.3.8.7 and above), a regular montage with minimum dimensions of 2 × 2 was collected with the designated overlaps in *x* and *y* at the data acquisition magnification. The beam-image-shift tiling information (ImageShift entry under each item section in the image metadata file ‘.mdoc’ file) was obtained on the fly. The shift in the *x* direction to achieve the frame overlap of 15% in *X* was retrieved by calculating the difference in image shift between tile 1 (PieceCoordinates of 0 0 0) and tile 3 (PieceCoordinates of 4608 0 0). The shift in the *y* direction to achieve the frame overlap of 10% overlap in *y* was retrieved by calculating the difference between tile 1 (PieceCoordinates of 0 0 0) and tile 2 (PieceCoordinates of 0 3516 0). The MultishotParams (*x*/*y* component of image-shift vector) was subsequently modified to reflect the tile montage image shift and saved under the SerialEM setting file (tutorial available on https://github.com/wright-cemrc-projects/cryoet-montage/). In SerialEM 4.1 beta (https://bio3d.colorado.edu/ftp/SerialEM/Beta-4.1-64/), the tile overlaps and MultishotParams can be directly modified via the regular Montage Setup Dialog window without the need to identify the beam-image-shift information via initial 2 × 2 montaging and to modify the setting file subsequently. We recommend SerialEM 4.1 for easier application of the MPACT scheme. The step-by-step setup can be found at https://github.com/wright-cemrc-projects/cryoet-montage/tree/main/SerialEM/.

#### MPACT cryo-tilt-series collection in SerialEM

The SerialEM macro corresponding to SerialEM 3.8 and above (stable release) or 4.1, directly exported from TomoGrapher, also available at https://github.com/wright-cemrc-projects/cryoet-montage/, was used to implement MPACT by acquiring overlapping tiles with designated overlaps to form a montage at each tilt with an additional translational shift and/or rotation shift to distribute the dose. Autofocusing was performed at each tilt and shifted along the orientation of the tilt axis 500 nm plus the maximum translational shift of the center of montage tile pattern (for example, 0.8 µm; Extended Data Fig. 8) away from the edge of the montage tile pattern. The total dose for each tile tilt series was 40–50% below the maximum dose the sample was able to tolerate before evidence of punctate bubbles. At the beginning of each grouped tilt, high-magnification/data acquisition tracking with a threshold of 5% of the FOV to acquire a new tracking reference and to iterate the alignment until the threshold was met (usually within one or two iterations), using the nearby autofocusing area, was performed. An additional lower intermediate magnification tracking on a larger FOV of the ROI was implemented when the tilt angles were above 30°, with a threshold of a difference greater than 10% (usually within one or two iterations). The tilt-series collection was paused when the iterations for convergence exceeded five times. Benchmarks were done using a 3 × 3 or 3 × 4 montage tile pattern and a dose-symmetric scheme running at ±60° with 3° increments (±51° with 3° increments for the neuron sample and A549 lamella grids; Fig. 2), groups of three tilts (original ‘Hagen scheme’ is group of one tilt) and a dose of ~2 e^−^/Å^2^ per tile per tilt for RSV-infected BEAS-2B (defocus range of −3, −3.5, −4, −4.5, −5, −5.5, −6 μm) and HeLa cells (defocus of −5 μm), and neurons on micropatterned grids (defocus of −5 μm), while a dose of 1.42 e^−^/Å^2^ per tile per tilt for A549 cell lamella (defocus of −8 μm) based on the dose tolerance measurements of each sample. The CDS counting mode and dose rate of 10 e^−^pixel^−1^s^−1^ over the sample were used on a K3 camera. The translational shift offsets followed the global spiral displacement (A_final_ = 1.5, period = 3, turns = 50, revolutions = 15 as input parameters to control the spiral size and resulting displacement offsets). The speed of collection varied with the size of the montage tile, the type of microscope, detector used and sample-dependent total dose. Benchmark collections were ~60–75 min on average and rendered nine sub-tilt series (3 × 3 tile pattern) that were stitched seamlessly to form one montaged tilt series. Translational spiral offsets and collection schemes (for example, bidirectional, dose symmetric and defocus) can be adjusted accordingly in the SerialEM macro. The SerialEM macro can be modified to adjust translational spiral offsets and collection schemes (for example, bidirectional, dose symmetric and defocus). The macro can be integrated into existing common automated data collection schemes using the function ‘Navigator Acquire at points’ in SerialEM^50^.

#### Montage tilt-series processing

All raw movie frames/fractions per acquisition (5,760 × 4,092, counting mode, 0.1–0.3 e^−^/Å^2^ per frame) were aligned and corrected for motion using MotionCor2 (ref. ^51^). For montage tilt series, two approaches were explored to make stitched montage tomograms. The total tiles per tilt were registered and stitched together using the designated beam coordinates supplied to the microscope as described above and with linear cross-correlation methods^52^ at each tilt angle. Despite the fringes on the edge, intrinsic low contrast and low dose received by cryo-tilt series, the regularity of the montage tiling pattern and sufficient overlaps with optimized tiling strategy consistently provided robust and automated seamless stitching without user intervention up to ±60°. Manual image alignment (MIDAS), implemented in the IMOD package^52^, was used to adjust piece coordinates and iteratively cross-correlate adjacent tiles at higher tilts when necessary. Fully stitched montage tilt series were binned by 2. Tilt-series alignment and tomographic reconstructions were performed as a single frame type using the IMOD Tomography package/Etomo with a final pixel size of 9.206 Å. In the absence of gold fiducials in the FIB-milled lamella, alignment of the tilt series was performed using patch tracking. CTF correction using strip-based ctfplotter and ctfphaseflip and dose-weighted filtering^39^ were applied to the aligned tilt series before tomogram reconstruction. For segmentation, the aligned tilt series were further binned 3× (final pixel size of 27.6 Å, binned 6×) before the tomogram reconstruction. Tomograms were either processed using fast edge-enhancing denoising algorithm based on anisotropic nonlinear diffusion implemented in TomoEED^53^ or Gaussian low-pass filtered to 80 Å for visualization. Tomograms of stitched montage tilt series (final pixel size of 27.6 Å) were annotated using convolutional neural networks implemented in the EMAN2 package^54^. Alternatively, the stitched montage tilt series could be stitched as a whole tilt stack, aligned and then reconstructed in the IMOD Tomography/Etomo, following the montage-frame workflow to avoid the second interpolation when assembling the montage at each tilt separately and being processed as a non-montaged dataset as described above. It should be noted that montage stitching per tilt before the assembly of stitched tilt series leads to a smaller reconstructed area in the *y* direction due to increased overlap areas (*y* × cosine (*α*)) at high tilts (Extended Data Fig. 5b). The reliable area in the stitched tomogram will be reduced given the known artifacts present in cryo-ET reconstructions^55^. This can be mitigated by slightly increasing the acquisition tile pattern size or positioning the ROI within the center of the regular montage array and away from tile corners or boundaries. Alternatively, tile stitching can be conducted within the tilt series (montage-frame alignment and reconstruction in IMOD^24^). Nevertheless, this may affect stitching automation with thicker samples due to varying overlaps. For the individual tile tilt series, motion-corrected frames were sorted to generate individual tile tilt series that were CTF estimated using CTFFIND4 (ref. ^33^). Tilt series that contained one or more inadequate projections (not properly tracked or failed CTF estimation) were discarded. Qualified individual tile tilt series were then aligned, CTF corrected with stripped-based ctfplotter and ctfphaseflip, dose-weighted filtered, and reconstructed into tomograms, similarly to stitched montage tilt series implemented in the IMOD package^56^. The Python script (available at https://github.com/wright-cemrc-projects/cryoet-montage/) or bash script (available at https://github.com/wright-cemrc-projects/cryoet-montage/) was used to automate the movie frame alignment, montage tile stitching, sub-tilt and montage tilt generation. The deconvolve CTF step^57^ was performed on the 2D images to enhance the visualization.

### Sub-tomogram averaging

For proof of concept, all averaging was done on unfiltered individual tile tilt/sub-tilt tomograms using Dynamo (v.1.1.511)^58^, following the steps reported previously^59,60^. Particles were either manually or geometrically picked from low-pass filtered sub-tilt tomograms binned 2× to a final pixel size of 9.206 Å. An initial alignment was done with 914 particles manually picked from one tomogram. The final average from this first run was low-pass filtered to 20 Å and used as the initial reference for a second run on a total of 20 tile tilt tomograms with particles picked either manually or geometrically using a crop-on-rings-along-the-path filament model. Each tomogram was considered as one independent alignment and average project at this stage before the later combination. The default soft-edged spherical mask (box size of 42 voxels) and smoothing mask were applied during the alignment and Fourier compensation during averaging, respectively. Duplicate particles were removed after the alignment with a tolerance of 82.8 Å (9 voxels). Poorly aligned particles were discarded based on the cross-correlation score in each alignment project and validated via Chimera visualization^61^. The upside-down particles (the RSV F-pair was aligned to point toward the interior of the viral particles likely due to a poor density) were manually flipped using ‘dynamo_table_flip_normals’ in Dynamo and aligned again with a restricted search range. After the second run, an additional iteration run was done using a smaller search range. Duplicates (a tolerance of 9 voxels) and particles with low correlation scores were discarded again after the visual validation with either ArtiaX^62^ in ChimeraX^63^ or the ‘Place Object*’* plugin^64^ in Chimera^61^. The refined particles and orientations from the last run were mapped back to the full unbinned tomograms (pixel size of 4.603 Å), re-cropped with a voxel box size of 128, followed by another round of iterative alignments with a soft-edged cylinder mask around the densities of the RSV F-pairs, and a slightly increased low-pass filter (corresponding to 10 Å) was used during the alignment. After removing duplicates (18 voxels) and low correlation points, the refined particles from individual alignment projects/tomograms (one alignment project per tomogram so far) were combined, re-cropped, and split into the even and odd half-sets. Another round of alignment with the same alignment parameters as the last run was performed on the half-sets. After the low cross-correlation-scored normalized particles were discarded, the sub-tomogram average of each half-set was used to calculate the FSC plot in simpleFSC in PEET^65^. The final average of the RSV F-pair was computed by re-averaging all particles of the full dataset of 13,021 particles and then low-pass filtered to 24 Å based on the FSC cutoff of 0.5 (19.9 Å at FSC of 0.143, no symmetry imposed). The picked particles that were in overdosed areas (at least twofold and above the regular dose tolerance, ≥130 e/Å^2^) were removed and the rest were reprocessed following the same steps to generate the half-sets and final average. The final sub-tomogram average (final pixel size of 4.603 Å, unbinned) was reconstructed from 9,332 particles, and low-pass filtered to 22 Å based on the FSC cutoff of 0.5 (18.5 Å at FSC of 0.143). The overdosed F-pair particles (*n* = 3,689) were reprocessed following the same steps, of which the final density map was low-pass filtered to 34 Å based on the FSC cutoff of 0.5 (33.5 Å and 25.6 Å at FSC cutoff of 0.5 and 0.143, respectively). The same number of non-overdosed F-pair particles (*n* = 3,689) were picked out of the total pool of non-overdosed F-pairs (*n* = 9,332), processed following the same steps. The final density map was low-pass filtered to 28 Å based on the FSC cutoff of 0.5 (26.5 Å and 22.1 Å at FSC cutoff of 0.5 and 0.143, respectively). The atomic crystal model of pre-fusion F trimer (PDB 4JHW)^66^ was modeled into the filtered electron density map of the average generated from the overdosed removed particles (*n* = 9,332) in Chimera.

### TomoGrapher development

Simulations of the tiled montage imaging and electron beam exposures on a sample were developed as a collection of C# classes built on the Unity 3D engine (version 2020.3.20f1). The simulation represents an array of M × N × O volumes called voxels as a sample interacted by an electron beam. The canvas ‘stage’ comprises 150 × 150 voxels (*X* = *Y* = 150, *Z* = 1, total extent of 10 × 10 × 0.2 μm) with the center butterfly ROI spanning 5 × 4 μm on the imaging *XY* plane. An interactive GUI describes parameters of the imaging including sampling pixel spacing, illuminated area of the beam on the camera, the tiling pattern of the beam, tilt ranges and translations defined by spiral offsets. A complete run of a simulation iterates through each tilt increment across the range, and ray traces from a sampling position of the illuminated area in the direction of the beam to find intersections on the stage of voxel volumes. Voxels intersected by the rays are aggregated in a set, and each voxel in the set has its total dose incremented once per beam. The viewer provides a real-time animation of the beam shifts, tilting of the stage and overall exposure at each voxel. TomoGrapher release versions and source code are available at https://github.com/wright-cemrc-projects/cryoet-montage/.

### Statistics and reproducibility

Experiments in Fig. 2a–d and Extended Data Fig. 7a–g were performed independently three times over *n* = 5 various frozen labeled mammalian cell lamellae over three different grids with similar results. Experiments in Fig. 2e–j were performed independently over *n* = 96 primary *D. melanogaster* neurite targets over *n* = 19 different grids. Figures 1 and 2b and Extended Data Fig. 6a–e were performed independently three times over *n* = 5 RSV-infected cell periphery sites over three different grids with similar results. Experiments from Extended Data Figs. 1f–h and 2b were performed three times independently. Experiments from Extended Data Figs. 9a–e and 10b,c were performed three times independently over *n* = 50 RSV virus sites for montage cryo-ET.

### Reporting summary

Further information on research design is available in the Nature Portfolio Reporting Summary linked to this article.

## Online content

Any methods, additional references, Nature Portfolio reporting summaries, source data, extended data, supplementary information, acknowledgements, peer review information; details of author contributions and competing interests; and statements of data and code availability are available at 10.1038/s41592-023-01999-5.

## Supplementary information

Supplementary Information Supplementary Fig. 1, Table 1 and glossary and abbreviations.

Reporting Summary

Supplementary Video 1 Illustration of a typical simulation run in TomoGrapher. The ‘Image Shift Pattern’ and ‘Tilt Strategy’ panels are where the user adjusts a tilt-series collection scheme and applies user-specific translational shift to spread the dose. The bottom ‘Simulation’ panel supports various viewing angles, simulation operation and export of dose accumulation values in a csv file and SerialEM macro based on the parameters set up in the GUI. The SerialEM macro could be directly used in the batch montage tilt series acquisition. The source code and details are available at https://github.com/wright-cemrc-projects/cryoet-montage/.

Supplementary Video 2 In situ montage cryo-electron tomography of the lamella from a native A549 cell. The full tomographic volume and 3D segmentation shown in Fig. 1d are revealed when the movie slices through in *Z*. The corresponding cellular organelles, nucleus, nuclear pore complex, mitochondria, Golgi, rough endoplasmic reticulum and lipid droplets are labeled. Scale bar, 1 μm.

Supplementary Video 3 Montage cryo-electron tomography of primary *D. melanogaster* neurons grown on a mask-free micropatterned cryo-TEM grid. The movie slices through and back in *Z* of the 3 × 4 tomographic volume and segmentation, colored as in Fig. 2i–j to show the architecture of microtubules (green), mitochondria (dark blue with calcium granules in yellow), ribosomes (light pink) and ribosome-associated-like vesicles (darker cyan). Scale bar, 1 μm.

Supplementary Video 4 A 3 × 3 cryo-montage tilt series of the RSV-infected BEAS-2B cell after motion correction, tile stitching and tilt-series alignment are performed, as shown in Extended Data Fig. 9a. The movie shows the aligned tilt series from the tilt range of −60° to 60°. The clear spiral in-plane movement of the montage and individual tiles, introduced by the translational offset for dose distribution, is observed. Scale bar, 1 μm.

Supplementary Software 1 MATLAB scripts to generate piece coordinate files for stitching. When the dataset is collected using SerialEM 3.8 above and 4.0 stable release, the script ‘coordinate_mpact_SerialEM.m’ shall be used. The scripts are available at both https://github.com/wright-cemrc-projects/cryoet-montage/ and https://github.com/JaeEYang/MPACT_bashscripts/.

Supplementary Software 2 MATLAB scripts to generate piece coordinate files for stitching. When the dataset is collected using SerialEM 4.1, the script ‘coordinate_mpact_SerialEM4_1.m’ shall be used. The scripts are available at both https://github.com/wright-cemrc-projects/cryoet-montage/ and https://github.com/JaeEYang/MPACT_bashscripts/.

Supplementary Software 3 MATLAB scripts to generate piece coordinate files for stitching. Running either script will generate a list of piece coordinate files per tilt used for the subsequent stitching/blending. The output is in .txt format. Run ‘changename.sh’ afterwards in the same directory to get .pl format files. The scripts are available at both https://github.com/wright-cemrc-projects/cryoet-montage/ and https://github.com/JaeEYang/MPACT_bashscripts/.

Supplementary Software 4 Bash script to stitch and generate a montage tilt series. Run the bash script ‘blendstitching_tiltcompensated.sh’ after placing the bash script in the folder where the motion-corrected movie stacks per tilt are. The script will guide the user through the messages. In the end, a stitched montage tilt-series stack is generated. Each montaged tile stack per tilt is also sorted into individual folders based on the tilt angle along with the corresponding piece coordinate file. The user could intervene to adjust the stitching if the automated stitching performs less than ideally. The script is available at both https://github.com/wright-cemrc-projects/cryoet-montage/ and https://github.com/JaeEYang/MPACT_bashscripts/.

Supplementary Software 5 Bash script to sort and generate individual tile tilt series. Run the bash script ‘split_tiletiltseries.sh’ after placing the bash script in the folder where the motion-corrected movie stacks per tilt are. The script will guide the user through the messages. In the end, individual tile per tilt will be sorted into individual folders and a tile tilt-series stack will be generated. The script is available at both https://github.com/wright-cemrc-projects/cryoet-montage/ and https://github.com/JaeEYang/MPACT_bashscripts/.

## Data Availability

All relevant data are available from the corresponding author upon reasonable request. A set of raw frames (Extended Data Fig. 9a; a representative 3 × 3 montage cryo-tilt series) is provided as a demo dataset and available to download at https://github.com/wright-cemrc-projects/cryoet-montage/tree/main/Tutorial/ for demonstration of pre-processing steps including montage tilt-series generation and stitching. Sub-tomogram averages have been deposited in the Electron Microscopy Data Bank (https://www.emdataresource.org/) under accession numbers EMD-40308 (Extended Data Fig. 10; sub-tomogram average of total RSV F-pair particles picked from individual tile tilt/sub-tilt montage cryo-ET tomogram tiles) and EMD-40307 (Extended Data Fig. 10; sub-tomogram average of overdose removed RSV F-pair particles picked from individual tile tilt/sub-tilt montage cryo-ET tomogram tiles).
