# Supplementary material for: Correlative montage parallel array cryo-tomography for in situ structural cell biology
Source: Nat Methods. 2023 Sep 18;20(10):1537–43. doi: 10.1038/s41592-023-01999-5 (PMC10555823; doi:10.1038/s41592-023-01999-5)
Supplement: Supplementary file 1 — Supplementary Fig. 1, Table 1 and glossary and abbreviations. [file 41592_2023_1999_MOESM1_ESM.pdf]

nature methods

Article https://doi.org/10.1038/s41592-023-01999-5

Correlative montage parallel array cryo-tomography for in situ structural cell biology

In the format provided by the authors and unedited

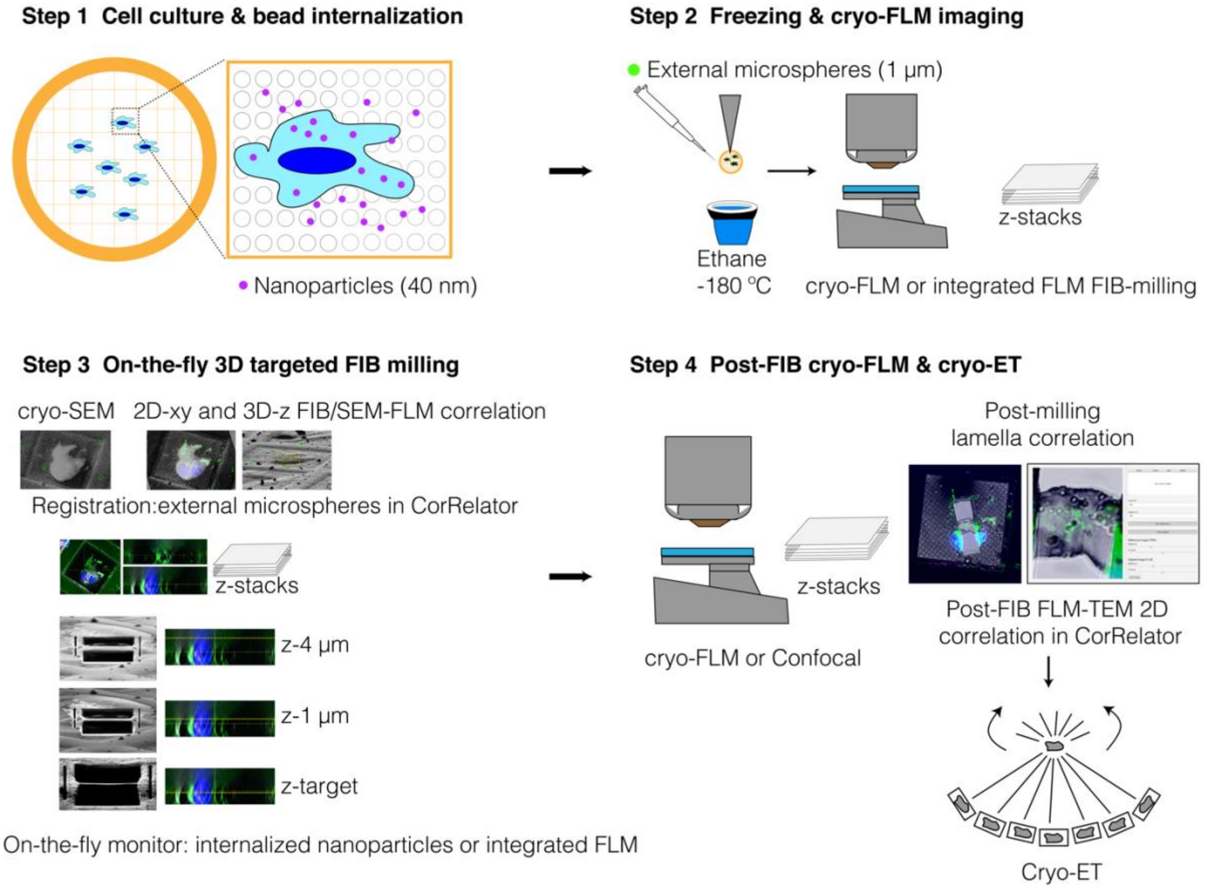

Supplementary Figure 1. Correlative 3D cryo-FLM-FIB-ET workflow. Overview of the correlative 3D cryo-FLM-FIB-ET workflow using two fiducial markers: 1 μm external fluorescent microspheres for initial registration and cell-internalized 40 nm nanoparticles for on-the-fly 3D refined correlation during cryo-FIB milling. **Step 1.** Low-toxic fluorescent nanoparticles (40 nm, pink) incubated with cultured cells were internalized. **Step 2.** Fluorescent microspheres (1 μm) were added prior to plunge freezing and grids were then imaged under an external cryo-FLM system to acquire z-stacks of regions of interest (ROIs). **Step 3.** The same grid was loaded onto and imaged with a dual-beam cryo-FIB-SEM system. Using the 3DCT Toolkit and CorRelator, cryo-FLM z-stacks and 2D cryo-SEM and cryo-FIB images of ROIs were correlated for milling using 1 μm microspheres (FIB view, green circles) in x, y, and z. The placement of the milling boxes was refined on-the-fly based on the relative positions of the nanoparticles (pink) and signal of interest (green) as the thinning proceeded to compensate for the movement of lamella due to the milling action. **Step 4.** The same FIB-milled lamella could return to the cryo-FLM or cryo-Confocal system to acquire a new z-stack cryo-FLM images for post-FIB-milling correlation. If the dual-beam system is equipped with an integrated fluorescent objective lens, the presence of the beads and ROI could be captured as well as the lamella is being thinned down to 200 nm, including the initial pre-FIB-milled FLM and final FIB-milled FLM stacks. The lamella was then loaded into a cryo-TEM system. Precise correlation between

2D cryo-FLM and TEM images of the lamella in CorRelator was conducted, and montage or regular tilt series were collected with SerialEM.

Tilt Strategy Factors	Position 1 (x/y, 35/103)	Position 2 (x/y, 35/87)	Position 3 (x/y, 58/103)	Position 4 (x/y, 58/87)
**No translation (a)**	61.0 $\pm$ 6.3	82.3 $\pm$ 1.1	96.7 $\pm$ 5.4	130.1 $\pm$ 17.3
**No translation lower dose by 30% (b)**	43.1 $\pm$ 4.2	57.6 $\pm$ 0.7	67.7 $\pm$ 3.6	107.7 $\pm$ 6.5
**Translation (c)**	55.1 $\pm$ 9.5	71.2 $\pm$ 8.5	76.03 $\pm$ 8.2	92.7 $\pm$ 6.2
**Translation lower dose by 30% (d)**	41.6 $\pm$ 5.7	50.0 $\pm$ 5.7	53.2 $\pm$ 5.8	64.9 $\pm$ 4.3
**Translation with x-axial correction (e)**	59.2 $\pm$ 12.5	83.3 $\pm$ 10.6	81.1 $\pm$ 12.3	109 $\pm$ 7.1
**Translation with x-axial correction lower dose by 30% (f)**	41.2 $\pm$ 8.7	58.2 $\pm$ 7.2	57.7 $\pm$ 8.6	76.3 $\pm$ 4.8

Supplementary Table 1. Simulated dose accumulation in $e^-/Å^2$/voxel at specified voxels in four sampling areas (radius of 10 x 10 x 1 voxel around the central sampling points 1 to 4, X and Y voxel positions specified, as indicated in **Extended Data Fig. 4**) of six different collection schemes with the same benchmarked default 3X3 montage tile pattern in *TomoGrapher*. The mean and standard deviation were calculated. The Tilt Strategy parameters is also specified in Extended Data Fig. 4 (3X3 tile pattern, pixel size of 4.603 Å, beam size of 3.15 μm, from -60° to 60° tilts with a 3° increment, 1 or 0.7 $e^-/Å^2$ per tile per tilt).

Glossary and Abbreviations

1. General

Montage Parallel Array Cryo-Tomography (MPACT)
Under parallel illumination, the beams are packed in a regular array tile pattern either rectangular or square (m x n) with defined overlaps between beam tiles to form a montage at each tilt angle during tilt series acquisition. The final 3D reconstruction is called a tomogram.

Region of Interest (ROI)
The sample region targeted for data acquisition.

Sub-Tomogram Averaging (STA)
A process where equivalent volumes of an object of interest are extracted from one or multiple full tomograms and averaged together to produce a structure at improved resolution.

2. Beam illumination and image frame

Pre-exposed area
The sample area being illuminated by the beam yet falling outside the camera frame.

Full Illuminated Area (FIA)
The sample area being illuminated by the beam.

Field of View (FOV)
The sample area being captured as non-blank contents on the camera frame.

3. Tilt Strategy/Translational shift

Amplitude initial
The starting point and relative distance from the original (0,0) in a polar coordinate system, together with Amplitude final to determine the scale.

Amplitude final
The ending point and relative distance from the original (0,0) in the first cycle in a polar coordinate system, together with Amplitude initial to determine the scale.

Revolutions and Turns
Determine the growth of the spiral curve, namely the velocity as the point moving and turns. Larger Revolutions and turns lead to faster velocity.

Periods
The distance between two branches in a spiral along the same direction. It determines the overall shape of the trajectory.

All four parameters above together determine the size of the spiral.
