# Supplementary material for: Correlative montage parallel array cryo-tomography for in situ structural cell biology
Source: Nat Methods. 2023 Sep 18;20(10):1537–43. doi: 10.1038/s41592-023-01999-5 (PMC10555823; doi:10.1038/s41592-023-01999-5)
Supplement: Supplementary file 2 — Reporting Summary [file 41592_2023_1999_MOESM2_ESM.pdf]

nature research

Corresponding author(s): E.R. Wright, manuscript ID: NMETH-BC48073
Last updated by author(s): June 20, 2023

Reporting Summary

Nature Research wishes to improve the reproducibility of the work that we publish. This form provides structure for consistency and transparency in reporting. For further information on Nature Research policies, see our Editorial Policies and the Editorial Policy Checklist.

Statistics

For all statistical analyses, confirm that the following items are present in the figure legend, table legend, main text, or Methods section.

n/a	Confirmed	
☐	☒	The exact sample size (*n*) for each experimental group/condition, given as a discrete number and unit of measurement
☐	☒	A statement on whether measurements were taken from distinct samples or whether the same sample was measured repeatedly
☒	☐	The statistical test(s) used AND whether they are one- or two-sided<br>*Only common tests should be described solely by name; describe more complex techniques in the Methods section.*
☒	☐	A description of all covariates tested
☐	☒	A description of any assumptions or corrections, such as tests of normality and adjustment for multiple comparisons
☐	☒	A full description of the statistical parameters including central tendency (e.g. means) or other basic estimates (e.g. regression coefficient) AND variation (e.g. standard deviation) or associated estimates of uncertainty (e.g. confidence intervals)
☒	☐	For null hypothesis testing, the test statistic (e.g. *F*, *t*, *r*) with confidence intervals, effect sizes, degrees of freedom and *P* value noted<br>*Give P values as exact values whenever suitable.*
☒	☐	For Bayesian analysis, information on the choice of priors and Markov chain Monte Carlo settings
☒	☐	For hierarchical and complex designs, identification of the appropriate level for tests and full reporting of outcomes
☒	☐	Estimates of effect sizes (e.g. Cohen's *d*, Pearson's *r*), indicating how they were calculated

Our web collection on statistics for biologists contains articles on many of the points above.

Software and code

Policy information about availability of computer code

Data collection	SerialEM 3-8-7 64 bit, SerialEM 4-0-15 64-bit, SerialEM 4-1-0beta11_64, Leica LAS X, Leica LAS X THUNDER
Data analysis	Unity 2021.3.22f1, Python 3.8.5, IMOD 4.11.11, MatLab 2020b (MathWorks), 3DCT 2.2.2, UCSF MotionCor2 1.3.1, PRISM 9 (GraphPad), Dynamo (v1.1.511), TomoGrapher, Python, bash, and MatLab scripting developed for this manuscript (https://github.com/wright-cemrc-projects/cryoet-montage) and CorRelator (https://github.com/wright-cemrc-projects/corr)

For manuscripts utilizing custom algorithms or software that are central to the research but not yet described in published literature, software must be made available to editors and reviewers. We strongly encourage code deposition in a community repository (e.g. GitHub). See the Nature Research guidelines for submitting code & software for further information.

Data

Policy information about availability of data

All manuscripts must include a data availability statement. This statement should provide the following information, where applicable:

- Accession codes, unique identifiers, or web links for publicly available datasets
- A list of figures that have associated raw data
- A description of any restrictions on data availability

EMD-40308 (Extended Data Fig 10, top panel, STA of RSV-F pair and M picked from MPACT), EMD-40307 (Extended Data Fig 10, bottom panel, STA of overdose-removed/non-overdosed, RSV-F pair and M picked from MPACT); A set of raw frames (Extended Data Fig 9, a representative 3X3 montage cryo-tilt series) is provided as the Demo data set and available via https://github.com/wright-cemrc-projects/cryoet-montage/ tree/main/Tutorial, to download for demonstration of pre-processing steps including montage tilt series generation and stitching. The EMDB data depositions will be available to the journal and reviewers upon request directly to EMDdataResource site

Field-specific reporting

Please select the one below that is the best fit for your research. If you are not sure, read the appropriate sections before making your selection.

☒ Life sciences ☐ Behavioural & social sciences ☐ Ecological, evolutionary & environmental sciences

For a reference copy of the document with all sections, see nature.com/documents/nr-reporting-summary-flat.pdf

Life sciences study design

All studies must disclose on these points even when the disclosure is negative.

Sample size	3D targeted cryo-FLM-FIB-MPACT was performed independently four times over four different grids on three different samples (A549 cells, RSV-infected A549 cells, HeLa cells), independently three times on primary Drosophila melanogaster neurons grown on maskless-micropatterned grids. MPACT was performed independently three times over two different samples (RSV-infected HeLa cells and RSV-infected BEAS-2B cells) to collect 2D correlative montage tilt series and individual tile tilt series used for sub-tomogram averaging (STA). In total, 20 selected individual tile tilt series were used for averaging.
Data exclusions	For sub-tomogram averaging, individual tile tilt series collected via the benchmark 3x3 montage-cryo ET dose-symmetric scheme with failed CTF estimation (CTFFIND4), or no viral particles were excluded.
Replication	Shift displacement of individual tiles and stitched tilt series using spiral translation only were calculated from n = 8 stitched 3x3 or 3x4 tilt series and individual tile tilt series, n = 3 for the translation plus rotation 2x2 montage tilt series. CTF estimation and defocus determination using both IMOD/ctfplotter and CTFFIND4 were done using n = 7 stitched 3x3 montage tilt series and individual tile tilt series.
Randomization	The grids that were imaged under cryo-FLM-FIB/SEM-montage cryoET were selected randomly from duplicates of 2 to 3 grids of the same samples.
Blinding	No blinding of subjects was involved.

Reporting for specific materials, systems and methods

We require information from authors about some types of materials, experimental systems and methods used in many studies. Here, indicate whether each material, system or method listed is relevant to your study. If you are not sure if a list item applies to your research, read the appropriate section before selecting a response.

Materials & experimental systems

n/a	Involved in the study	
☒	☐	Antibodies
☐	☒	Eukaryotic cell lines
☒	☐	Palaeontology and archaeology
☐	☒	Animals and other organisms
☒	☐	Human research participants
☒	☐	Clinical data
☒	☐	Dual use research of concern

Methods

n/a	Involved in the study	
☒	☐	ChIP-seq
☒	☐	Flow cytometry
☒	☐	MRI-based neuroimaging

Eukaryotic cell lines

Policy information about cell lines

Cell line source(s)	ATCC
Authentication	Authenticated through ATCC upon purchasing
Mycoplasma contamination	No contamination
Commonly misidentified lines (See ICLAC register)	N/A

Animals and other organisms

Policy information about studies involving animals; ARRIVE guidelines recommended for reporting animal research

Laboratory animals	Drosophila melanogaster (fruit fly) was used for the extraction of primary neurons form third-instar larvae. These neurons were then cultured on cryo-EM grids and imaged.
Wild animals	N/A
Field-collected samples	N/A
Ethics oversight	N/A

Note that full information on the approval of the study protocol must also be provided in the manuscript.
